# Correction: Doing what matters in times of stress: No-nonsense meditation and occupational well-being in COVID-19

**DOI:** 10.1371/journal.pone.0305180

**Published:** 2024-06-04

**Authors:** Justine Van de Velde, Katia Levecque, Bert Weijters, Steven Laureys

## Notice of republication

This article was republished on May 21, 2024, to add trial registration to the abstract, and S1 (TREND Statement Checklist) and S2 (Study protocol and data management plan) files have been added. Please download this article again to view the correct version. The originally published, uncorrected article and the republished, corrected articles are provided here for reference.

## Supporting information

S1 File Originally published, uncorrected article. (PDF)

S2 File Republished, corrected article. (PDF)
