# Supplementary material for: Doing what matters in times of stress: No-nonsense meditation and occupational well-being in COVID-19
Source: PLoS One. 2023 Nov 1;18(11):e0292406. doi: 10.1371/journal.pone.0292406 (PMC10619828; doi:10.1371/journal.pone.0292406)
Supplement: S2 File — (PDF) [file pone.0292406.s002.pdf]

FACULTEIT PSYCHOLOGIE EN PEDAGOGISCHE WETENSCHAPPEN

NEDERLANDS/DUTCH

SPECIFIEK ETHISCH PROTOCOL

VOOR WETENSCHAPPELIJK ONDERZOEK AAN DE FACULTEIT PSYCHOLOGIE EN PEDAGOGISCHE WETENSCHAPPEN VAN DE UNIVERSITEIT GENT

Versie d.d. 18 maart 2015

Ethische Commissie, Faculteit Psychologie en Pedagogische Wetenschappen, Universiteit Gent, Henri Dunantlaan 2, 9000 Gent

VERZOEK TOT ADVIES VAN DE ETHISCHE COMMISSIE MET BETREKKING TOT HET HIERNA VERMELDE ONDERZOEKSVOORSTEL:

TITEL VAN HET ONDERZOEK:

Meditatieproject Vlaanderen: effecten van meditatie op het welzijn van leerkrachten

NAAM VAN DE ONDERZOEKER(S):

Prof. Dr. Katia Levecque

TELEFOONNUMMER: 09 264 63 66

FACULTEIT: Psychologie en Pedagogische Wetenschappen

VAKGROEP: Werk, Organisatie en Samenleving (PP09), Henri Dunantlaan 2, 9000 Gent

ENGELS/ENGLISH

SPECIFIC ETHICAL PROTOCOL

FOR SCIENTIFIC RESEARCH AT THE FACULTY OF PSYCHOLOGY AND EDUCATIONAL SCIENCES OF GHENT UNIVERSITY

Version d.d. 18 maart 2015

Ethical Committee, Faculty of Psychology and Educational Sciences, Ghent University, Henri Dunantlaan 2, 9000 Gent

REQUEST TO THE ETHICAL COMMITTEE FOR ADVICE CONCERNING THE FOLLOWING RESEARCH PROPOSAL

TITLE OF THE RESEARCH PROJECT:

Meditation project Flanders: effects of meditation on teachers' well-being

NAME OF THE RESEARCHER(S):

Prof. Dr. Katia Levecque

PHONE: 09 264 63 66

FACULTY: Psychologie en Pedagogische Wetenschappen

DEPARTMENT: Werk, Organisatie en Samenleving (PP09), Henri Dunantlaan 2, 9000 Gent

UNIVERSITEIT GENT

Prof. Dr. Martin Valcke

TELEFOONNUMMER: 09 264 86 75

FACULTEIT: Psychologie en Pedagogische Wetenschappen

VAKGROEP: Onderwijskunde (PP06), Henri Dunantlaan 2, 9000 Gent

Prof. Dr. Martin Valcke

PHONE: 09 264 86 75

FACULTY: Psychologie en Pedagogische Wetenschappen

DEPARTMENT: Onderwijskunde (PP06), Henri Dunantlaan 2, 9000 Gent

Prof. Dr. Lieven Annemans

TELEFOONNUMMER: 09 332 60 37

FACULTEIT: Geneeskunde en Gezondheidswetenschappen

VAKGROEP: Volksgezondheid en Eerstelijnszorg, Corneel Heymanslaan 10, ingang 42 – verdieping 4, 9000 Gent

Prof. Dr. Lieven Annemans

PHONE: 09 332 60 37

FACULTY: Geneeskunde en Gezondheidswetenschappen

DEPARTMENT: Volksgezondheid en Eerstelijnszorg, Corneel Heymanslaan 10, ingang 42 – verdieping 4, 9000 Gent

IS ER EEN FINANCIËLE SPONSOR VOOR DIT PROJECT?

Neen

IS THERE A FINANCIAL SPONSOR FOR THIS PROJECT?

No

MAAKT HET ONDERZOEK DEEL UIT VAN EEN SAMENWERKING DIE DE FACULTEIT OVERSTIJGT? ZO JA, GEEF AAN WELKE CENTRA ER NOG BIJ BETROKKEN ZIJN.

Dit onderzoek kadert in een multidisciplinair project waarbij onderzoekers verbonden aan de faculteit Psychologie en Pedagogische Wetenschappen samenwerken met onderzoekers verbonden aan de faculteit Geneeskunde en Gezondheidswetenschappen om het effect van een interventie (namelijk meditatie) op het welzijn en leervermogen van leerlingen en op het welzijn van leerkrachten en leerlingen in basisscholen in kaart te brengen.

Het aanvraagdossier voor het onderzoekgedeelte dat zich richt op de kinderen, werd ingediend bij de Ethische Commissie van de Faculteit Geneeskunde en Gezondheidswetenschappen (dd. 23/10/2020).

Het aanvraagdossier voor het onderzoeksgedeelte dat zich richt op de leerkrachten, wordt ingediend bij de Ethische Commissie van de Faculteit Psychologie en Pedagogische Wetenschappen.

IS THE PROJECT PART OF ANY COOPERATION BEYOND THE FACULTY? IF SO, SPECIFY THE INSTITUTIONS INVOLVED.

This study is part of a multidisciplinary project in which researchers affiliated with the Faculty of Psychology and Educational Sciences are collaborating with researchers affiliated with the Faculty of Medicine and Health Sciences to identify the effect of an intervention (i.e., meditation) on students' well-being and learning ability and on the well-being of teachers and students in elementary school.

The application for the research part focusing on children was submitted to the Ethics Committee of the Faculty of Medicine and Health Sciences (dated 23/10/2020).

The application for the research portion focusing on teachers is submitted to the Ethics Committee of the Faculty of Psychology and Educational Sciences.

Het initiatief voor de interventie kwam vanuit de ondersteuningsnetwerken WAN, Meetjesland, Kasterlinden en Brussel Centrum. Deze netwerken zullen ook de interventie organiseren en opvolgen.

The initiative for the intervention originated from the educational support networks WAN, Meetjesland, Kasterlinden and Brussel Centrum. These networks will also organize and monitor the intervention.

GEEF EEN KORTE SAMENVATTING VAN HET ONDERZOEK (200 À 400 WOORDEN MAXIMUM EN VERSTAANBAAR VOOR MENSEN NIET GESPECIALISEERD IN DE MATERIE); LEG HET ACCENT OP WAT FEITELIJK EN CONCREET GAAT GEBEUREN TIJDENS HET ONDERZOEK (EN DUS NIET OP DE THEORETISCHE ACHTERGROND), VAN REKRUTERING TOT RAPPORTERING.

- Het initiatief tot introductie van meditatie in scholen van het lager onderwijs gaat initieel uit van een zorgcoördinator uit het WAN-netwerk (Wetteren, Aalst, Ninove). Er werd een oproep gedaan via de pers tot vrijwillige deelname aan het project. Leerkrachten uit Vlaamse en Brusselse basisscholen konden zich inschrijven. Bij deze oproep werd reeds gecommuniceerd dat de effecten van meditatie op het welzijn en leervermogen van leerlingen en op het welzijn van leerkrachten onderwerp zouden uitmaken van een wetenschappelijk onderzoek.
- Alle leerkrachten die deelnemen aan de interventie krijgen een korte opleiding in de meditatietechniek (train de trainer). Zij zullen vervolgens de meditatietechniek aan hun leerlingen aanleren. De interventie duurt 1,5 jaar en start in januari 2021. De interventie bestaat eruit dat de leerkracht dagelijks, samen met de leerlingen, enkele minuten mediteert. Er wordt voor elke leerkracht een draaiboek voorzien De leerkrachten zullen bijkomend ondersteund worden door zorgcoördinatoren en andere begeleiders uit de officiële ondersteuningsnetwerken van de scholen.
- Alle leerkrachten die zich hebben ingeschreven voor deelname aan het mediatieproject worden uitgenodigd tot deelname aan het onderzoek. Deelname wordt aangemoedigd, maar blijft op vrijwillige basis. De instemming tot deelname aan het onderzoek zal via informed consent worden geregistreerd. Om de effecten van de meditatie op het functioneren van de leerkrachten na te gaan, plannen we een pre-meting in januari 2021.

PROVIDE A SUMMARY OF THE RESEARCH. FORMULATE YOUR SUMMARY IN A WAY THAT IS UNDERSTANDABLE FOR PEOPLE WHO ARE NOT FAMILIAR WITH THE SUBJECT MATTER. DO NOT EMPHASIZE THE THEORETICAL BACKGROUND, BUT RATHER WHAT WILL HAPPEN IN PRACTICE DURING THE RESEARCH, FROM RECRUITMENT TO REPORTING.

- The initiative to introduce meditation in primary schools initially came from an educational support coordinator employed by the WAN network (Wetteren, Aalst, Ninove). An appeal was made through the press for voluntary participation in the project. All teachers working in Flemish and Brussels elementary schools could register. With this call, it was already communicated that the subject of a scientific study would be the effects of meditation on the well-being and learning ability of pupils and on the well-being of teachers.
- All teachers participating in the intervention will receive a short training in the meditation technique (train the trainer). They will then teach the meditation technique to their students. The intervention will last 1.5 years and start in January 2021. The intervention consists of the teacher meditating daily, together with the students, for a few minutes. A script will be provided for each teacher. The teachers will additionally be supported by educational support coordinators and other facilitators from the official support networks of the schools.
- All teachers who have registered to participate in the mediation project are invited to participate in the study. Participation is encouraged but remains voluntary. Consent to participate in the study will be recorded via informed consent. To ascertain the effects of meditation on teachers' well-being, we plan a pre-measurement in January 2021.

- Deze online bevraging zal fungeren als een basismeting van hun functioneren voorafgaand aan de start van de interventie. Na deze eerste meting zal er in maart 2021 een tussentijdse online bevraging uitgevoerd worden. In juni 2021 zal aan de leerkrachten een laatste online vragenlijst worden aangeboden. Tussen de metingen door wordt tweewekelijks een korte priksurvey georganiseerd waarbij we aan de hand van twee à drie vragen polsen naar de ervaringen van de leerkrachten en de deelnemers de mogelijkheid geven op eventuele problemen aan te kaarten of feedback te geven (tevredenheid met de ondersteuning vanuit de ondersteuningsnetwerken, beheersing van de meditatietechniek, algemene gezondheid van de leerkracht).
- De controlegroep voor dit project wordt gevormd door leerkrachten en leerlingen die behoren tot scholen uit het scholennetwerk van de initiatiefnemers, maar die geen vraag tot participatie aan het mediatieproject hebben ingediend. Ook bij deze controlegroep wordt deelname door leerkrachten aangemoedigd, maar blijft het op vrijwillige basis. De instemming tot deelname aan het onderzoek zal via informed consent worden geregistreerd. Bij de controlegroep worden identieke vragenlijsten afgenomen als in de interventiegroepen, met uitzondering van vragen die gerelateerd zijn aan de interventie. De afname van de vragenlijst gebeurt op dezelfde manier en op dezelfde momenten als bij de interventiegroepen. De deelnemers van de controlegroep krijgen de mogelijkheid om vanaf september 2021 alsnog in het mediatieproject in te stappen.

HOUDT HET ONDERZOEK GEVAREN IN VOOR DE GEZONDHEID VAN DE PARTICIPANTEN? INDIEN JA, HEBT U OOK EEN AANVRAAG GEDAAN BIJ EEN MEDISCH ETHISCH COMITE?

Neen

- This online survey will act as a baseline measurement of their well-being prior to the start of the intervention. After this initial measurement, an interim online survey will be conducted in March 2021. A final online survey will be offered to teachers in June 2021. In between the measurements, a short prick-survey will be organized every two weeks in which we will poll the experiences of the teachers by means of two or three questions and give the participants the opportunity to raise any problems or give feedback (satisfaction with the support from the support networks, mastery of the meditation technique, general health of the teacher).
- The control group for this project is formed by teachers and students who belong to schools in the initiators' school network, but who have not submitted a request to participate in the mediation project. Also in this control group, teacher participation is encouraged but remains voluntary. Consent to participate in the study will be recorded via informed consent. In the control group, identical questionnaires will be administered as in the intervention groups, except for questions related to the intervention. The questionnaire will be administered in the same manner and at the same times as in the intervention groups. Starting in September 2021, the participants of the control group will have the opportunity to learn the meditation technique.

DOES THE RESEARCH PROJECT PRESENT ANY THREATS TO THE PARTICIPANTS' PHYSICAL OR MENTAL HEALTH? ARE THERE REASONS TO CONSIDER A REVIEW BY A MEDICAL ETHICAL COMMITTEE?

No

GAAT HET HIER OM PARTICIPANTEN MET VOORAF GEKENDE PROBLEMEN? INDIEN JA, GEEF AAN OF HET ONDERZOEK KAN INTERFEREREN MET DE PROBLEMATIEK EN WELKE VOORZORGEN U IN DIT GEVAL ZULT NEMEN.

Neen

ZIJN DE DEELNEMERS MEERDERJARIGEN DIE ONBEKWAAM ZIJN HUN TOESTEMMING TE VERLENEN?

Neen

ZIJN DE DEELNEMERS MINDERJARIG (JONGER DAN 18 JAAR)?

Het aanvraagdossier voor het onderzoekgedeelte dat zich richt op de kinderen, werd ingediend bij de Ethische Commissie van de Faculteit Geneeskunde en Gezondheidswetenschappen. De deelname aan het onderzoek door de kinderen gebeurt vanuit de leerplandoelstellingen die passen bij de eindtermen met betrekking tot welzijn. In Vlaanderen is er expliciet aandacht voor het ontwikkelen van deze dimensie in kinderen. Het onderzoek sluit dus aan op de opdracht van de school. De aanvrager verantwoordelijk voor het deelonderzoek bij de kinderen (Prof. Dr. Lieven Annemans) zal voor fases 2 en 3 actief geïnformeerde toestemming vragen aan de ouders of voogden van de deelnemende kinderen.

Gegevens die tot op heden werden verzameld zonder de uitdrukkelijke toestemming van de ouders of voogden zullen niet gebruikt worden in het deelonderzoek bij de leerkrachten. Tot op heden hebben de aanvragers verantwoordelijk voor het deelonderzoek bij de leerkrachten (Prof. Dr. Katia Levecque en Prof. Dr. Martin Valcke) nog geen gegevens ontvangen uit het deelonderzoek bij de kinderen (o.l.v. Prof. Dr. Lieven Annemans).

DO THE PARTICIPANTS HAVE ANY KNOWN DIFFICULTIES? ARE THEY A VULNERABLE POPULATION?

No

ARE THE PARTICIPANTS ADULTS WHO ARE INCOMPETENT TO GIVE THEIR CONSENT?

No

ARE THE PARTICIPANTS MINORS (UNDER 18 YEARS OF AGE)?

The application dossier for the research portion focusing on the children was submitted to the Ethics Committee of the Faculty of Medicine and Health Sciences. The children's participation in the research is based on the curriculum objectives that fit the final attainment levels related to well-being. In Flanders, there is an explicit focus on developing this dimension in children. The research is therefore in line with the school's assignment. The applicant responsible for the children's substudy (Prof. Dr. Lieven Annemans) will actively seek informed consent from the parents or guardians of the participating children for phases 2 and 3.

Data collected to date without the explicit consent of the parents or guardians will not be used in the teacher substudy. To date, the applicants responsible for the teachers' substudy (Prof. Dr. Katia Levecque and Prof. Dr. Martin Valcke) have not received any data from the children's substudy (led by Prof. Dr. Lieven Annemans).

Indien deze gegevens in de toekomst toch gedeeld zouden worden met de onderzoekers van het project bij de leerkrachten, zal dit beperkt worden tot de gegevens van kinderen wiens ouders of voogden actief hun toestemming verleenden. Deze gegevens zullen binnen het onderzoek bij de leerkrachten enkel gebruikt wanneer die geaggregeerd zijn op klasniveau (gemiddelden).

Should these data be shared in the future with the researchers of the teachers' project, this will be limited to the data of children whose parents or guardians actively gave their consent. This data will be used within the teacher research only when aggregated at the classroom level (averages).

ZAL MISLEIDING GEBRUIKT WORDEN IN HET ONDERZOEK?

Neen

WILL DECEPTION BE USED DURING THE RESEARCH PROJECT?

No

OP WELKE MANIER WORDEN DE PARTICIPANTEN GEÏNFORMEERD OVER DE RESULTATEN VAN HET ONDERZOEK? WELKE DEBRIEFING IS VOORZIEN?

Alle deelnemende leerkrachten krijgen, indien ze dat wensen, het eindrapport opgestuurd. Via de priksurveys zullen ze ook op de hoogte gebracht worden van tussentijdse bevindingen. De onderzoeksbevindingen zullen verder verspreid worden via wetenschappelijke publicaties

WILL THE PARTICIPANTS BE INFORMED ABOUT THE RESULTS OF THE RESEARCH PROJECT? WILL THERE BE A DEBRIEFING?

All participating teachers, if they wish, will be sent the final report. Through the prick surveys, they will also be informed of interim findings. The research findings will be further disseminated through scientific publications.

WORDEN STUDENTEN INGESCHAKELD VOOR DE REKRUTERING VAN DEELNEMERS, VOOR DE DATA-VERZAMELING EN/OF -VERWERKING? ZO JA, WELKE STAPPEN WORDEN ONDERNOMEN OM STUDENTEN HIEROP VOOR TE BEREIDEN?

Ja

- Een masterstudent verbonden aan de faculteit Psychologie en Pedagogische Wetenschappen zal een masterproef schrijven op basis van de data die bij de leerkrachten gedurende dit project verzameld wordt.
- De rekrutering van deelnemers gebeurt niet door de masterstudent, de student zal enkel gebruikmaken van data-analytische technieken om de onderzoeksvraag van diens masterproef te beantwoorden.

WILL STUDENTS BE INVOLVED IN THE RECRUITMENT OF PARTICIPANTS, DATA COLLECTION OR DATA PROCESSING? IF SO, WHAT STEPS ARE TAKEN TO PREPARE AND GUIDE STUDENTS?

Yes

- A master's student affiliated with the Faculty of Psychology and Educational Sciences will write a master's thesis based on the data collected from teachers during this project.
- The recruitment of participants is not done by the master's student, the student will only use data analytical techniques to answer the research question of the master's thesis.

- De student is gedurende de opleiding tot bachelor/master of science in bedrijfspsychologie en personeelsbeleid voorbereid op het toepassen van deze data-analytische technieken en wordt hierin verder begeleid door onderzoekers verbonden aan het project.
- De student krijgt toegang tot een deel van de dataset die enkel die data bevat die relevant zijn voor het beantwoorden van de onderzoeksvraag van de masterproef. Contactgegevens van deelnemers worden niet gedeeld met de student.

WAT VOORZIET U INZAKE DATA-MANAGEMENT, TIJDENS EN NA HET ONDERZOEK? GELIEVE TE FOCUSSEN OP ASPECTEN DIE RELEVANT KUNNEN ZIJN VOOR DE ETHISCHE COMISSIE. HOE WORDEN DE DEELNEMERS OP DE HOOGTE GEBRACHT VAN UW PLANNEN?

- De data bij de leerkrachten wordt verzameld via een online bevraging. De communicatie gebeurt via een email-adres dat door de leerkracht wordt gekozen. De data verzameld tijdens de basismeting, tussentijdse meting, eindmeting en priksurveys zullen aan elkaar gekoppeld worden op basis van een unieke code. Die koppeling gebeurt door een *Trusted Third Party*.
- Het databestand wordt op een afgesloten folder op de sharepoint server van de UGent geplaatst en is enkel toegankelijk door de onderzoekers van deze aanvraag. Ook de informed consentformulieren en de informatiebrief die aan de deelnemers worden gegeven, zullen in deze folder worden opgeslagen.
- Alle data, inclusief de ondersteunde informatie m.b.t. de opzet en de uitvoering van het onderzoek, blijven vijf jaar na de publicatie van een onderzoeksartikel bewaard op de sharepoint server (zie beleidskader https://www.ugent.be/nl/onderzoek/datamanagement/beleidskader-rdm.pdf).
- De deelnemers worden voorafgaand aan hun deelname op de hoogte gebracht van de plannen omtrent data-management via een informatiebrief en geven expliciet hun toestemming tot het verzamelen, verwerken en tijdelijk bewaren van de surveyresultaten via een informed consentformulier.

- The student is prepared during the undergraduate/Master of Science program in industrial and organizational psychology to apply these data analytical techniques and is further guided in this by researchers associated with the project.
- The student will only have access to the portion of the dataset that is relevant to answering the research question of the master's thesis. Contact information of participants will not be shared with the student.

WHAT ARE YOU PREPARING FOR DATA MANAGEMENT, DURING AND AFTER THE RESEARCH? PLEASE FOCUS ON ASPECTS THAT MAY BE RELEVANT TO THE ETHICS COMMITTEE. HOW WILL PARTICIPANTS BE INFORMED OF YOUR PLANS?

- Data from teachers is collected via an online survey. Communication will be through an email address chosen by the teacher. The data collected during baseline measurement, interim measurement, final measurement and prick surveys will be linked together based on a unique code. That linking will be done by a Trusted Third Party.
- The data file will be placed on a locked folder on the UGent sharepoint server and will only be accessible by the researchers that perform this project. The informed consent forms and information letter given to participants will also be stored in this folder.
- All data, including supporting information related to the design and conduct of the research, will be retained on the sharepoint server for five years after the publication of a research article (see policy https://www.ugent.be/nl/onderzoek/datamanagement/beleidskader-rdm.pdf).
- Participants are informed of data management plans via an information letter prior to participation and explicitly give their consent to the collection, processing and temporary retention of survey results via an informed consent form.

VOORZIET U IN HET HUIDIGE STADIUM VAN HET PROJECT ANDERE PROBLEMEN MET DE ALGEMENE ETHISCHE UITGANGSPUNTEN ZOALS BESCHREVEN IN HET ALGEMENE ETHISCHE PROTOCOL?

Neen

IN THE CURRENT STATE OF THE RESEARCH PROJECT, DO YOU EXPECT OTHER DIFFICULTIES CONCERNING THE GENERAL ETHICAL PRINCIPLES AS WRITTEN DOWN IN THE GENERAL ETHICAL PROTOCOL?

No

VERKLARING

Ik verklaar de volledige verantwoordelijkheid van het hierboven vermelde project op mij te nemen en bevestig dat voor zover de huidige kennis het toelaat, de gegeven inlichtingen met de werkelijkheid overeenstemmen.

Ik verklaar ook het algemene ethische protocol voor wetenschappelijk onderzoek FPPW-UG doorgenomen te hebben en dit te onderschrijven voor alle punten waarvoor geen opmerkingen gemaakt werden onder 6A-H.

Indien tijdens het verloop van het project ethische problemen rijzen die niet door dit verzoek gedekt worden, zal ik opnieuw contact opnemen met de ethische commissie.

DECLARATION

I declare to take the full responsibility of the project mentioned above and confirm that the information given is consistent with the facts as known at this very moment.

I also declare to have read and agree with the General Ethical Protocol for scientific research of the Faculty of Psychology and Educational Sciences of Ghent university.

If, during the project, ethical problems arise that are not covered by this request, I will contact the Ethics Committee again.

Onderzoeker 1/Researcher 1

Datum/Date: 14 December 2020

Naam/Name: Prof. Dr. Katia Levecque

Handtekening/Signature:

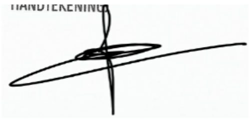

Onderzoeker 2/Researcher 2

Datum/Date: 14 December 2020

Naam/Name: Prof. Dr. Martin Valcke

Handtekening/Signature:

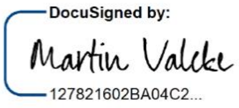

Onderzoeker 3/Researcher 3

Datum/Date: 14 December 2020

Naam/Name: Prof. Dr. Lieven Annemans

Handtekening/Signature:

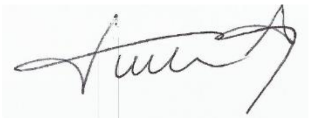

Data Management Plan

Admin details

Project Name Meditatieproject Vlaanderen: Effecten van Meditatie op het Welzijn en Functioneren van Leerkrachten - 20210412_DMP_Meditatieproject_Vlaanderen_Leerkrachten

Principal Investigator / Researcher Justine Van de Velde, Katia Levecque, Martin Valcke, and Lieven Annemans

Description The project is part of a multidisciplinary investigation into the effects of meditation. The goal of this project is to examine how the practice of meditation affects the well-being and functioning of Flemish elementary school teachers and their pupils. This project will primarily focus on the meditation effects on teacher well-being, while other investigators will focus on the effects of meditation on the pupils. To investigate the effects of meditation on teacher well-being, an intervention study will be conducted, where teachers learn a specific meditation technique. Participants will be asked to maintain a regular meditation (with their pupils) and will be asked to fill in multiple questionnaires between January 2021 and June 2022.

Institution Ghent University

Administrative Data

Date of first version

30/11/2020

Date of last update

12/04/2021

1. Data Collection

1.1 What data will you collect or create?

Types of collected data

This project regarding the effects of meditation on teacher well-being will collect data using online questionnaires at different points in time between January 2021 and June 2022.

Data formats

The data from the online surveys will be downloaded as .xlsx-files to permit statistical analyses using SPSS or R. For analyses in SPSS, .csv-files will be created.

1.2 How will the data be collected or created?

The .xlsx-files with raw data and the created .csv-files will be stored by date and project name (Meditatieproject Vlaanderen Leerkrachten 2021: MVL21), using the following template: *YYYYMMDD_MVL21_pretest*. This will

allow the researcher to retrieve the correct file version later on in the project.

Data cleaning is done in SPSS using syntax. The syntax files are stored in order to see the stepwise changes of the data cleaning and to correct any mistakes if necessary. For this reason, the raw data is still stored. The data is pseudonymized.

Data are stored on the UGent Shares, which takes regular backups.

2. Data Documentation and Metadata

2.1 How will you document the data?

The principal investigator will document the descriptive and contextual information needed to interpret and use the data in the future (i.e. the general research design, the context of the data collection, the methodological procedures followed during data collection and analysis, etc.). This will be done in the following ways:

- The principal investigator will write a logbook (MS Word), documenting which steps are taken in the project and why. This logbook will be updated regularly.
- When SPSS is used, codebooks will be constructed (and updated as needed), containing more information about the variables in the dataset (i.e. names, labels, descriptions, units of measurement, references to the questions in the survey, etc.)
- All steps taken during the data analysis will be documented in annotated syntax files.
- Any published research article will include a methodology section documenting the data gathering and analysis.

3. Ethical and legal issues

3.1 How will you manage any ethics and confidentiality issues?

Ethics

For the collection, use, preservation, and sharing of the data, the 'General Ethical Protocol for Scientific Research at the Faculty of Psychology and Educational Sciences of Ghent University' will be used as a guide. Also, the advice of the Ethical Committee will be asked.

Personal data

The collected data will contain individual identification codes. These are needed to link the data of the different measurement moments to the right participant. This way, we can document the evolution of participants throughout the intervention period.

Informed consent

Each participant will have to sign an informed consent to be allowed to participate. With this informed consent, the participant explicitly gives the researchers permission to collect, analyze, and temporarily store the data.

For the parallel project regarding the effects of meditation on pupil well-being, informed consent will be asked from the parents or legal guardians of the underage pupils.

Protecting identities

The identities of the participants will be protected using pseudonymization (the names will be replaced by identification codes). There will be an encrypted key file containing the link between the codes and the identities of the participants. This file will only be available to a trusted third party during the project. This trusted third party will not have access to the data collected by the surveys. The principal investigators will not have access to this key file, making it impossible for them to link the identification codes or the collected data to the names or contact details of the participants.

Transfer of data

To date, the principal investigators responsible for examining how meditation affects teacher well-being have not received any data resulting from the parallel project on pupil well-being. If and when any data regarding the well-being of pupils will be shared with the researchers of the teacher project, this transfer of data will be limited to the data of pupils whose parents or legal guardians provided informed consent. Additionally, the data will be aggregated to group data prior to transfer.

The collected data regarding the effects of meditation on the well-being of teachers will only be accessible for third parties when granted permission by the principal investigators. Prior to any transfer, all data will be anonymized.

At the moment, there are no plans to share the data with researchers who are not currently involved in the project.

3.2 How will you manage intellectual property rights issues?

Ghent University is the owner of the data collected during this project.

Only anonymized data will be eligible for sharing. Sharing will only take place with the explicit permission of the responsible researchers.

4. Data Storage and Backup during Research

4.1 How will you store and backup data during research?

Data are stored on UGent shares and shared with the principal investigators only. UGent shares are protected with version control and regular backups are made.

4.2 How will you ensure that stored data are secure?

The data on the UGent Shares are only accessible to the principal investigators. When leaving the computer unattended, it is logged off.

The shares are controlled using Active Directory, which allows only certain people to have access to the data.

When collaborating with other researchers or students, the anonymized data will only be transferred when these collaborators have signed a Non-

Disclosure Agreement. Sharing the data will only be possible for research purposes. If a transfer is necessary, it will be done using BelNet FileSender.

5. Data Selection and Preservation after Research

5.1 Which data should be retained for preservation and/or sharing?

- All research material collected by the researcher will be preserved for at least five years. This is in accordance with the UGent policy on research data management.
- Key files connecting participant identification codes with their identities will be destroyed after the five-year preservation period.

5.2 What is the long-term preservation plan for the selected datasets?

After the PhD-project, all the digital research material will be transferred to the shared network drive maintained by the promotor of the project. Data will be anonymized where possible.

6. Data Sharing

6.1 Are any restrictions on data sharing required?

- The data collected during this project only contains participant identification codes. The key file connecting these codes with the identity of the participants will not be shared with the principal investigators, only the trusted third party has access to it.
- Participants (or their parents/legal guardians) are required to sign an informed consent form containing a section in which they explicitly give permission to share their data with other researchers after anonymization.
- There are currently no plans to share the data with other researchers who are not involved in the project. Even if the responsible researchers decide to share any data, only anonymized data will be eligible for sharing.

6.2 How will you share data selected for sharing?

There are currently no plans to share data, however, if data are shared:

- Data will only be shared upon request and if permission is given by the responsible researchers (i.e. the doctoral student and the supervisors).
- Data will only be shared if permission is given by the participants or their parents or legal guardians (through informed consent).
- Data will be anonymized.

7. Responsibilities and Resources

7.1 Who will be responsible for data management?

The principal investigators are responsible for data management.

If any of the principal investigators should leave UGent, the meta-data and the data are still accessible for the other responsible researchers involved in the project.

7.2 Will you need additional resources to implement your DMP?

No further resources are needed.
