# Supplementary material for: Doing what matters in times of stress: No-nonsense meditation and occupational well-being in COVID-19
Source: PLoS One. 2023 Nov 1;18(11):e0292406. doi: 10.1371/journal.pone.0292406 (PMC10619828; doi:10.1371/journal.pone.0292406)
Supplement: S1 Table — (DOCX) [file pone.0292406.s003.docx]

| **S1 Table.**  *Sample Size (n) by Condition and Time per Dimension of Well-Being* | | | |
| --- | --- | --- | --- |
| Well-being measure | January | March | June |
| Emotional Well-Being | | | |
| Perceived stress |  |  |  |
| Control condition | 38 | 27 | 21 |
| Intervention condition | 199 | 112 | 76 |
| Emotional exhaustion |  |  |  |
| Control condition | 38 | 27 | 21 |
| Intervention condition | 199 | 112 | 75 |
| Negative affect |  |  |  |
| Control condition | 37 | 27 | 21 |
| Intervention condition | 199 | 110 | 74 |
| Positive affect |  |  |  |
| Control condition | 37 | 27 | 21 |
| Intervention condition | 199 | 110 | 74 |
| Cognitive Well-Being | | | |
| Concentration problems |  |  |  |
| Control condition | 37 | 27 | 21 |
| Intervention condition | 198 | 109 | 74 |
| Physical Well-Being | | | |
| Musculoskeletal problems |  |  |  |
| Control condition | 37 | 27 | 21 |
| Intervention condition | 199 | 110 | 74 |
| Sleep problems |  |  |  |
| Control condition | 37 | 27 | 21 |
| Intervention condition | 199 | 110 | 74 |
|  | | | |
