# Supplementary material for: Doing what matters in times of stress: No-nonsense meditation and occupational well-being in COVID-19
Source: PLoS One. 2023 Nov 1;18(11):e0292406. doi: 10.1371/journal.pone.0292406 (PMC10619828; doi:10.1371/journal.pone.0292406)
Supplement: S2 Table — (DOCX) [file pone.0292406.s004.docx]

| **S2 Table.**  *Well-Being at Baseline: Independent Samples T-Test Based on Condition (0 = Control Condition, 1 = Intervention Condition) and Based on Completion (0 = Noncomplete, 1 = Complete)* | | | | | |
| --- | --- | --- | --- | --- | --- |
| Well-being measure | Condition | |  | Completion | |
|  | t | df |  | t | df |
| Emotional Well-Being | | | | | |
| Perceived stress | -0.59^n.s.^ | 235 |  | -0.25^n.s.^ | 235 |
| Emotional exhaustion | -1.13 ^n.s.^ | 235 |  | 0.29 ^n.s.^ | 235 |
| Negative affect | -0.62 ^n.s.^ | 234 |  | -0.78 ^n.s.^ | 234 |
| Positive affect | 1.90 ^n.s.^ | 234 |  | 0.59 ^n.s.^ | 234 |
| Cognitive Well-Being | | | | | |
| Concentration problems | 0.34 ^n.s.^ | 233 |  | -0.85 ^n.s.^ | 233 |
| Physical Well-Being | | | | | |
| Musculoskeletal problems | -1.08 ^n.s.^ | 234 |  | -0.42 ^n.s.^ | 234 |
| Sleep problems | -2.37^*^ | 234 |  | -1.15 ^n.s.^ | 234 |
| ^*^ *p* < .05; n.s. = not significant. | | | | | |
