# Supplementary material for: Doing what matters in times of stress: No-nonsense meditation and occupational well-being in COVID-19
Source: PLoS One. 2023 Nov 1;18(11):e0292406. doi: 10.1371/journal.pone.0292406 (PMC10619828; doi:10.1371/journal.pone.0292406)
Supplement: S3 Table — (DOCX) [file pone.0292406.s005.docx]

| **S3 Table.**  *Model Fit Indices for the Configural Invariant, Metric Invariant, and Scalar Invariant Models.* | | | | | | |  |
| --- | --- | --- | --- | --- | --- | --- | --- |
| Well-being measure | BIC | RMSEA | CFI | TLI | SRMR | χ²(df) | |
| Emotional Well-Being | | | | | | | |
| Perceived stress |  |  |  |  |  |  | |
| Configural invariance | 2266.16 | .05 | 0.99 | 0.98 | .03 | 25.54(15)^n.s.^ | |
| Metric invariance | 2246.71 | .05 | 0.99 | 0.98 | .04 | 27.96(19) ^n.s.^ | |
| Scalar invariance | 2225.24 | .03 | 1.00 | 0.99 | .04 | 28.37(23) ^n.s.^ | |
| Emotional exhaustion |  |  |  |  |  |  | |
| Configural invariance | 3042.81 | .04 | 0.99 | 0.99 | .04 | 20.43(15) ^n.s.^ | |
| Metric invariance | 3026.66 | .04 | 0.99 | 0.99 | .06 | 26.15(19) ^n.s.^ | |
| Scalar invariance | 3016.94 | .05 | 0.98 | 0.98 | .07 | 38.30(23) ^n.s.^ | |
| Negative affect |  |  |  |  |  |  | |
| Configural invariance | 2616.86 | .02 | 1.00 | 1.00 | .05 | 16.50(15) ^n.s.^ | |
| Metric invariance | 2599.83 | .02 | 1.00 | 0.99 | .06 | 21.33(19) ^n.s.^ | |
| Scalar invariance | 2583.48 | .03 | 1.00 | 0.99 | .05 | 26.83(23) ^n.s.^ | |
| Positive affect |  |  |  |  |  |  | |
| Configural invariance | 2231.83 | .04 | 0.99 | 0.98 | .04 | 20.38(15) ^n.s.^ | |
| Metric invariance | 2214.08 | .04 | 0.99 | 0.99 | .06 | 24.49(19) ^n.s.^ | |
| Scalar invariance | 2196.13 | .03 | 0.99 | 0.99 | .07 | 28.39(23) ^n.s.^ | |
| Cognitive Well-Being | | | | | | | |
| Concentration problems |  |  |  |  |  |  | |
| Configural invariance | ─ ^a^ | ─ ^a^ | ─ ^a^ | ─ ^a^ | ─ ^a^ | ─ ^a^ | |
| Metric invariance | ─ ^a^ | ─ ^a^ | ─ ^a^ | ─ ^a^ | ─ ^a^ | ─ ^a^ | |
| Scalar invariance | ─ ^a^ | ─ ^a^ | ─ ^a^ | ─ ^a^ | ─ ^a^ | ─ ^a^ | |
| Physical Well-Being | | | | | | | |
| Musculoskeletal problems |  |  |  |  |  |  | |
| Configural invariance | 4503.00 | .06 | 0.98 | 0.96 | .04 | 28.33(15) ^n.s.^ | |
| Metric invariance | 4486.12 | .06 | 0.98 | 0.96 | .05 | 33.31(19) ^n.s.^ | |
| Scalar invariance | 4471.83 | .06 | 0.98 | 0.96 | .05 | 40.88(23) ^n.s.^ | |
| Sleep problems |  |  |  |  |  |  | |
| Configural invariance | ─ ^a^ | ─ ^a^ | ─ ^a^ | ─ ^a^ | ─ ^a^ | ─ ^a^ | |
| Metric invariance | ─ ^a^ | ─ ^a^ | ─ ^a^ | ─ ^a^ | ─ ^a^ | ─ ^a^ | |
| Scalar invariance | ─ ^a^ | ─ ^a^ | ─ ^a^ | ─ ^a^ | ─ ^a^ | ─ ^a^ | |
| *Note.* BIC = Bayesian Information Criterion; RMSEA = Root Mean Square Error of Approximation; CFI = comparative fit index; TLI = Tucker-Lewis Index; SRMR = Standardized Root Mean Square Residual.  ^a^ Measurement invariance was not tested since this is a single-item measure.  n.s. = not significant. | | | | | | |  |
