# Supplementary material for: Correction: Doing what matters in times of stress: No-nonsense meditation and occupational well-being in COVID-19
Source: PLoS One. 2024 Jun 4;19(6):e0305180. doi: 10.1371/journal.pone.0305180 (PMC11149866; doi:10.1371/journal.pone.0305180)
Supplement: S1 File — (PDF) [file pone.0305180.s001.pdf]

PLOS ONE

RESEARCH ARTICLE

Doing what matters in times of stress: No-nonsense meditation and occupational well-being in COVID-19

Justine Van de Velde[1]*, **Katia Levecque**[1], **Bert Weijters**[1], **Steven Laureys**[2,3,4]

1 Department of Work, Organization, and Society, Ghent University, Ghent, Belgium, **2** Coma Science Group, GIGA Consciousness Research Unit, University and University Hospital of Liège, Liège, Belgium, **3** CERVO Brain Research Center, Laval University, Québec, Canada, **4** Consciousness Science Institute, Hangzhou Normal University, Hangzhou, Zhejiang, China

* justine.vandevelde@ugent.be

Check for updates

OPEN ACCESS

Citation: Van de Velde J, Levecque K, Weijters B, Laureys S (2023) Doing what matters in times of stress: No-nonsense meditation and occupational well-being in COVID-19. PLoS ONE 18(11): e0292406. https://doi.org/10.1371/journal.pone.0292406

Editor: Pedro J. Ramos-Villagrasa, Universidad de Zaragoza, SPAIN

Received: June 14, 2023

Accepted: September 19, 2023

Published: November 1, 2023

Copyright: © 2023 Van de Velde et al. This is an open access article distributed under the terms of the Creative Commons Attribution License, which permits unrestricted use, distribution, and reproduction in any medium, provided the original author and source are credited.

Data Availability Statement: Data cannot be shared publicly because of its sensitive nature. Data are available for researchers who meet the criteria for access to confidential data (contact via welinjevel_leraren@ugent.be).

Funding: The author(s) received no specific funding for this work.

Competing interests: The authors have declared that no competing interests exist.

Abstract

While the COVID-19 pandemic challenged the general public's health and well-being, it exacerbated the pre-existing well-being issues in the educational sector in many countries. Mindfulness-based interventions are often applied to protect and promote occupational well-being. To investigate how the well-being benefits of these interventions arise, we selected one accessible technique that is used in most of them: focused attention meditation. In the middle of the COVID-19 pandemic, 199 teachers voluntarily practiced five to ten minutes of meditation together with their pupils, every morning for six months. We employed a three-wave longitudinal design to follow any changes in the meditating teachers' well-being and compared these changes to a waitlist control condition of 42 teachers. Three dimensions of well-being were measured at baseline, half-time, and post-intervention: emotional, cognitive, and physical well-being. Latent growth curve models revealed that the meditation technique not only improves well-being but also prevents the development of well-being problems. The practice of focused attention meditation resulted in improvements in emotional and physical well-being and prevented the development of cognitive well-being problems that were observed within the control condition. The effects were strongest for emotional and cognitive well-being and followed a linear trend. This paper shows that the well-being effects of mindfulness-based interventions are at least in part due to the focused attention meditation that is practiced in them. Occupational groups that experience emotional, cognitive, or physical well-being issues can benefit from a few minutes of focused attention meditation per day.

Introduction

While COVID-19 triggered mental health challenges worldwide [1], its impact on occupational performance and well-being varied across labor markets, sectors, professions, and organizations. In many countries, the impact on the educational sector was huge [2–4],

exacerbating already existing high levels of stress, burn-out, depression, and anxiety among teachers [5–7]. The causes of lower occupational well-being in teachers are diverse and include job insecurity, high workload, work-life imbalance, behavioral and mental problems in students, as well as high parental expectations [8]. To protect and promote occupational well-being, several interventions have already been implemented, including mindfulness and meditation. During the COVID-19 pandemic, the United Nations explicitly recommended meditation to their employees as "a great option to incorporate into one's routine to reduce anxiety and stress" and provided free audio-guided meditation exercises [9].

In the current paper, we add to the existing knowledge base by assessing the effect of a classroom meditation intervention on occupational well-being during COVID-19 times. Using a 3-wave panel study of 276 meditating teachers and comparing them to a control group, we can map the within-person changes in emotional, cognitive, and physical well-being experienced over the course of six months.

Mindfulness-based interventions and focused attention meditation

Research interest in mindfulness-based interventions has flourished over the last decades and has found increasing empirical support for the relationship between mindfulness and diverse psychological health and well-being outcomes. Although research interest originated in the clinical field, scholars have recently begun to study the effect of workplace mindfulness-based interventions on occupational health, well-being, and performance. In their recent critique of the existing literature, Jamieson and Tuckey [10] present an overview of a wide variety of mindfulness-based interventions set out in different work contexts and working populations [10]. Some of the most researched approaches include mindfulness-based stress reduction [11], mindfulness-based cognitive therapy [12], dialectical behavior therapy [13], and acceptance and commitment therapy [14]. By now, the benefits of these interventions are well established in the broader mindfulness literature (for a recent review, see [15]), and are published in discipline areas such as clinical psychology, health psychology, nursing, education, and social work.

Theoretically, the Transactional Model of Stress [16] helps to explain the psychological benefits. According to the model, the cognitive appraisal of a stressor and the available coping resources influence the resulting amount of experienced strain [17]. Meditation develops an emotion regulation strategy called "reappraisal", which entails a change in the perception of stressors as less threatening and/or coping resources as more adequate, thereby reducing psychological strain [18]. Theoretical explanations of the physical effects of meditation are more elusive [19], but research has suggested that effects are reached through relaxation responses of the sympathetic nervous system [20], which would prevent the development of physical symptoms [21].

A closer look at the mindfulness literature reveals a huge variety in the composition of techniques that make up mindfulness-based interventions. By combining several techniques in one intervention, the well-being benefits of each of them remain unclear. In the current study, we look into the well-being benefits of an intervention based on one technique only: focused attention meditation. This type of meditation is included in most mindfulness-based interventions, as it is considered to be an accessible starting point for meditation novices. The goal of focused attention meditation is to reside in a vigilant state of awareness by maintaining a mental focus on a particular object such as a mantra, a burning candle, a visual image, or a physical sensation (e.g., the breath). Whenever the mind wanders, practitioners calmly redirect their attention [22,23]. Through this mental training, practitioners learn to remain vigilant to distractions, to recognize when their attention has been attracted by these distractions, and to return their attention to the original object [24].

The present study

In the present study, we assess whether focused attention meditation is in itself an effective strategy for the promotion of occupational well-being. In our research approach, we combine a longitudinal design with a multidimensional perspective on occupational well-being, enabling us to tap into within-person changes in different dimensions of well-being over the course of six months. Our research question is threefold:

Q1) Does focused attention meditation affect different dimensions of occupational well-being?

Q2) Are the effects of focused attention meditation equally strong for different dimensions of occupational well-being?

Q3) Does the effect of focused attention meditation on different dimensions of occupational well-being change over time?

Multidimensional well-being. Given the typically low correlation between various dimensions of well-being [25,26], our objective in this paper is to tap into the potentially different effects of focused attention meditation on different dimensions of well-being. We do so by following Halbreich's [27] differentiation between emotional, cognitive, and physical dimensions of well-being.

The emotional well-being of teachers has been a concern around the world: for example, many of them suffer from high levels of stress and burn-out [28,29]. Preliminary evidence on focused attention meditation suggests potential benefits for emotional well-being [22,23]. However, all former research on focused attention meditation has focused on meditation practice outside the workplace. Evidence regarding mindfulness-based interventions in the workplace is more developed and may therefore be more helpful in guiding our expectations regarding the effect of focused attention meditation in occupational settings. Numerous studies have found reductions in psychological strain and emotional exhaustion [30,31]. The results for affect are less conclusive since it has been investigated to a lesser extent [30]: some studies have found a significant beneficial effect [32,33], whereas others have not [34,35].

Cognitive well-being is usually regarded as the ability to perform mental processes such as information processing, problem solving and attention, which are vital parts of a teacher's job [36]. Neuroscientists have investigated and registered the positive effect of focused attention meditation on cognitive functions such as sustained attention. Using neuroimaging techniques (e.g., fMRI, EEG), they have established that the practice leads to increased activation in many parts of the brain, including regions that are involved in the regulation of attention [19,37]. However, once again, these studies were not performed in workplace settings.

Physical well-being has received little attention in research on meditation. Subclinical physical and somatic health complaints such as neck pain, back pain, and sleep problems are prevalent among teachers [38,39], but have rarely been studied in relation to focused attention meditation. In a clinical setting, two studies showed the health-improving effects of focused attention meditation on chronic back and neck pain [40,41]. In the work context, Janssen and colleagues [30] outlined that previous studies barely used any symptom-focused outcome measures. Only one workplace study on focused attention meditation has investigated physical outcomes and has found increases in sleep duration in meditating employees [42]. The workplace study of Jennings and colleagues [43] reported decreases in daily physical symptoms including headaches, back pain, and nausea following a mindfulness-based intervention. Theoretically, physical and somatic complaints might benefit from focused attention meditation in at least two different ways. One way is by raising health and body awareness, leading to the

identification of symptoms and complaints at an early stage. In its turn, this might spur appropriate actions, such as stretching, changing seating positions, or taking time for recovery. A second way, seen from the perspective of health prevention, is that meditation might help to mobilize the body and to trigger relaxation prior to the occurrence of symptoms.

Taken together, the research on focused attention meditation's effects on emotional, cognitive, and physical well-being is limited, especially in occupational settings. The more elaborate research on the effects of mindfulness-based interventions on occupational well-being provides some direction for hypotheses:

Hypothesis 1: *Focused attention meditation benefits emotional*, *cognitive*, *and physical occupational well-being*.

Differential effects on well-being dimensions. To date, no studies on focused attention meditation have simultaneously investigated and compared the emotional, cognitive, and physical well-being of employees. However, a few meta-analyses have investigated the effects of mindfulness-based interventions. Goldberg and colleagues [44] performed a systematic review of 44 meta-analyses that exclusively looked into randomized controlled trials of mindfulness-based interventions (the 'gold standard design' in intervention research). Effect sizes varied depending on the type of control condition (i.e., active or waitlist control condition) and the time of comparison (i.e., at post-intervention or follow-up), but effect sizes were consistently larger for emotional dimensions of well-being than for physical dimensions of well-being. Other systematic reviews and meta-analyses into the effects of mindfulness-based interventions have presented a similar picture: strongest effects for indicators of emotional well-being, and smaller effect sizes for physical well-being [31,33,44]. The cognitive dimensions are not included in these reviews. Those studies investigating the cognitive dimension usually present small effect sizes of mindfulness-based interventions on attention-related indicators of the cognitive dimension [45,46]. No meta-analytic findings were available for the effect sizes associated with focused attention meditation.

Taking the above-described findings into account, we formulate the following hypothesis:

Hypothesis 2: *Focused attention meditation has stronger beneficial effects on emotional well-being compared to cognitive and physical well-being*.

Changes in well-being effects over time. Shapiro [47] suggested that the effects of meditation may change as practitioners' goals and expectations evolve. However, studies investigating these changes in effects are unsurprisingly scant, as this type of investigation poses high demands on study design. Cross-sectional meditation studies are performed very often, but only provide a snapshot, which inhibits the observation of changes in individual well-being over time. Longitudinal research offers a more promising approach, but most of the existing longitudinal studies end after six to eight weeks of meditation [33,48] and entail only a baseline and a post-intervention measurement [49]. Additionally, these studies focus exclusively on full mindfulness-based interventions. No research on potential changes in the effects of focused attention meditation exists to date.

The design of the present study allowed us to fill this gap by comparing the different patterns of change that were suggested by mindfulness-based intervention research. The first possibility is that the effect of meditation is linear. One study has explicitly tested different patterns and has only found evidence for a linear relationship between time and physical well-being: well-being gradually improved after a few days of mindfulness meditation practice and continued to improve at the same rate [50]. Second, the effect might be quadratic, with increasing returns over time: while the first sessions of mindfulness meditation only led to small improvements in well-being, the continued engagement in mindfulness meditation practices

over time resulted in larger, clinically significant improvements [49,51]. Third, the effect might start linear and then level off into a plateau [50]. This view is consistent with the idea of diminishing returns: at a certain level of experience, more meditation no longer results in further increases in well-being. To the best of our knowledge, this plateau effect of meditation has only been formulated hypothetically and has never been empirically tested.

Based on the limited empirical evidence on changes in the well-being effects of mindfulness-based interventions over time, we formulate three competing hypotheses on potential changes in the effects of focused attention meditation:

Hypothesis 3a: *The effects of focused attention meditation on occupational well-being follow a linear pattern.*

Hypothesis 3b: *The effects of focused attention meditation on occupational well-being follow a quadratic pattern.*

Hypothesis 3c: *The effects of focused attention meditation on occupational well-being follow a plateau pattern.*

Method

Procedure and participants

This study was part of a larger project that was set up by four employees working in four different educational support networks to assess the effect of an intervention encompassing daily focused attention meditation in the classroom in elementary schools. They initiated this project as a part of their job as educational support providers, the project itself did not acquire any additional funding. The intervention was initiated by an open call in the regional news in the Fall of 2020, inviting all teachers in all elementary schools in Flanders and Brussels to participate in the project. Participation in the meditation intervention was voluntary, not imposed by school boards, free of charge or financial incentive and entailed an engagement of daily classroom meditation sessions over the course of 18 months. The project focused on the meditation's well-being effects for teachers and students at the same time. Student well-being was part of the project's focus because recent research has shown an increase in well-being issues (e.g., autism spectrum disorder (ASD), attention-deficit hyperactivity disorder (ADHD), mood and anxiety disorders, and conduct disorders) among children and adolescents over the last decades [52]. The COVID-19 pandemic further increased these issues [53,54]. Therefore, one of the project's aims was to support the students by teaching them a new coping skill.

In this study, the effect of the focused attention meditation intervention on teacher occupational well-being was tested using three online self-report surveys over the first six months of the project, comparing an intervention condition with a waitlist control condition. Participation in the self-report surveys was voluntary in both groups and no monetary compensation was provided. The study was approved by the Ethical Committee of Ghent University. The participants of this study did not give written consent for their data to be shared publicly, so due to the sensitive nature of the research, the supporting data cannot be made publicly accessible.

In total, 276 teachers subscribed themselves to participate in the meditation project and were subsequently invited to participate in the intervention condition of this study as well. Eighty-seven teachers who did not participate in the meditation project were invited to participate in the control condition of this study. They were not informed that they were a member of a control condition in an effect study of meditation. The sole information provided to them was that they took part in a six-month study that monitors teachers' occupational well-being

during the COVID-19 pandemic. To avoid contamination between the two conditions, the control condition participants were recruited in schools that did not participate in the intervention condition. Additionally, the measures taken by the government in response to the pandemic gravely limited the amount of social contact between all people in general (e.g., (partial) lockdowns, restrictions on the number of people one could have contact with).

Both the intervention group and the control group were administered the same three online self-report surveys: a baseline survey in January 2021, a half-time survey in March 2021, and a post-intervention survey in June 2021. Participants who did not complete the baseline survey were excluded from the study and did not receive invitations to the second and third surveys. A trusted third party that was in no other way involved in this study kept the code file in which the participants could be identified. The authors of this manuscript did not have access to any information that could be used to identify participants, nor during, nor after data collection was completed.

Of the 276 project participants, 199 teachers in 84 different schools provided their informed consent and completed the baseline survey in January 2021 (response rate: 72.10%). Attrition in the intervention condition resulted in subsequent sample sizes of 129 in the half-time survey (response rate: 46.74%) and 92 at post-intervention (response rate: 33.33%). Informed consent and baseline data were collected for 42 control condition participants from 14 different schools (response rate: 48.28%). Response rates for the subsequent surveys were as follows: 29 completed the half-time survey (response rate: 33.33%), and 24 participated in the post-intervention survey (response rate: 27.59%). Non-response bias analyses showed no indications of selective attrition: attrition patterns were not related to any major sociodemographic characteristics or allocation to one of the two conditions (i.e., intervention condition or waitlist control condition). It was, however, related to individual experiences with COVID-19, such as getting infected with the virus or having a family member or friend who got infected.

Table 1 shows that nine in ten participants identified themselves as female. The majority of the sample was aged between 25 and 44 (65.98%), though the age ranged from 18 to 60 in the full sample. Baseline gender and age distributions in the control condition were not significantly different from the intervention condition, and quite accurately reflected the distribution in the population of elementary school teachers in Flanders (83.42% female, 57.28% between 25 and 44 years old). In Flanders, a teaching position requires successful higher education training. Jobs are well-protected by stringent labor laws and laws on occupational well-being. Teachers' remuneration is non-negotiable, follows governmental pay scales, and is guaranteed even during sick leave. Table 1 also shows that about half of the intervention condition participants had no experience with meditation before the start of the intervention, neither in their personal lives nor in their professional careers as a teacher. The other half had at least some experience with meditation in either their personal life or in their job as a teacher, or both.

Intervention

The intervention was initiated in January 2021. At that time, the COVID-19 pandemic forced governments to impose strict rules on social interactions. These rules changed regularly, but in general, most workplaces remained closed and remote work was obliged where possible. As an exception to the general rule, elementary schools remained open at all times and teachers were asked to stick to the normal curriculum as much as possible. Stringent rules for conduct, social contact, and hygiene were imposed to minimize the risk of COVID-19 infection. Teachers were asked to always wear a face mask, while pupils were not obliged. For safety reasons, entrance to schools was limited for external visitors. As a result, the intervention instructions were administered in one online instruction session (+/- 2 hours). The educational support

Table 1. Participant characteristics for the intervention condition, the control condition, and the total sample: Percentages.

Characteristic	Total Sample (n = 241)	Intervention Condition (n = 199)	Control Condition (n = 42)	χ^2(df)
Gender				
Male	9.13	8.54	11.91	0.47(1)$^{n.a.}$
Female	90.87	91.46	88.10	
Age, in years				
18–24	6.64	7.04	4.76	6.31(4)$^{n.a.}$
25–34	31.95	33.17	26.19	
35–44	34.03	35.68	26.19	
45–54	19.09	17.09	28.57	
55–60	8.30	7.04	14.29	
Meditation experience				
No experience	—[a]	50.25	—[a]	
Experience	—[a]	49.75	—[a]	
Compliance with meditation practices				
Participation in classroom meditation				
March 2021	—[a]	91.58	—[a]	
June 2021	—[a]	100.00	—[a]	
At-home practice				
March 2021	—[a]	46.32	—[a]	
June 2021	—[a]	47.44	—[a]	

Note. χ^2 = comparison of proportions between the intervention condition and the control condition.

n.s. = not significant.

[a] not measured in the control condition.

https://doi.org/10.1371/journal.pone.0292406.t001

networks that initiated the project were the only external visitors who were allowed physical access to the schools during the pandemic, and therefore they provided the participants with day-to-day support and advice regarding the meditation. The session was recorded and uploaded to the project's website, which also included other video and audio files that supported the meditation practice. Access to these materials was granted on school days and non-school days, though only for teachers and pupils participating in the meditation project to avoid contamination with the control condition. This approach established standardization in the training and support of all participants and also supported voluntary at-home practices for teachers and pupils.

The train-the-trainer instruction session was performed by an employee of one of the educational support networks who is an experienced meditator. The session started with practical information: participants were asked to organize one classroom meditation session at the beginning of every school day. The duration of the meditation session depended on the age of the pupils and varied between five and ten minutes. The teachers were tasked with guiding their pupils but were also recommended to join the pupils in meditation when the progress of their pupils allowed them to do so. Additionally, teachers were recommended to take up a regular at-home practice of 20 minutes per session.

The instruction session explained how to set up for the meditation session in three steps: (1) finding or creating a calm place where one will not be disturbed (e.g., closed door and curtains, dimmed lights, calming music), (2) sitting down on (a cushion on) the floor or in a chair with the feet planted firmly on the ground and a straight back, and (3) closing the eyes. Once these preparations were made, the meditation session could start. An audio file guided the

participants, explaining what to do at different points during the session. After a few slow, deep breaths, the breath is allowed to return to its natural rhythm, and the participants are instructed to focus on the physical sensation of the breath. During the session, the audio guide acknowledges that mental distractions are normal and that the purpose of meditation is not to avoid them, but to become aware of them and to learn how to return the focus to the breath. This procedure is followed until the end of the session.

As can be seen in Table 1, at the time of the second survey, nine out of ten teachers reported being able to participate in these classroom meditation sessions, and 46.32% of the participants regularly engaged in at-home practices as well. At post-intervention, all teachers participated in the classroom meditation sessions and 47.44% of the teachers supplemented the classroom meditation with at-home sessions.

Measures: Well-being

As already stated, well-being was measured by tapping into several dimensions: emotional well-being, cognitive well-being, and physical well-being. Emotional well-being was measured by three indicators: perceived stress, emotional exhaustion, and affect. Perceived stress was measured using the Perceived Stress Scale [55]. The items included "How often have you been upset because of something that happened unexpectedly?", and "How often have you felt that you were unable to control the important things in your life?". Answers ranged from 1 (*never*) to 5 (*very often*). The internal consistency of the scale was high and stable across time ($\alpha_{January}$ = .90, α_{March} = .88, α_{June} = .92). Emotional exhaustion was measured using the Maslach Burnout Inventory, adapted for use in populations of educators [56,57]. The items included "I feel worn out at the end of a working day", and "I feel tired as soon as I get up in the morning and see a new working day stretched out in front of me". Participants responded to these items using a scale ranging from 1 (*not at all*) to 5 (*very much*). Internal consistency of the scale was high and stable across time ($\alpha_{January}$ = .89, α_{March} = .90, α_{June} = .91). Affect was measured using the Positive and Negative Affect Scale (PANAS; [58]). Participants rated how often they felt positive emotions such as enthusiasm, pride, or excitement, as well as negative emotions such as shame, anger, or fear on a scale ranging from 1 (*not at all*) to 5 (*very much*). Internal consistency of both the positive affect and the negative affect scale was high and stable across time (positive affect: $\alpha_{January}$ = .87, α_{March} = .87, α_{June} = .90; negative affect: $\alpha_{January}$ = .81, α_{March} = .86, α_{June} = .87).

The indicator for the cognitive dimension of well-being was the frequency of concentration problems. This was measured by a single question tapping into the frequency in the past three months. Fisher and colleagues [59] found that single-item measures can be useful in measuring various constructs, especially when survey length poses a significant burden on respondents. Answers ranged from 1 (*never or almost never*) to 5 (*almost every day or every day*).

Finally, physical health was measured as the frequency of musculoskeletal problems and sleep problems. We used a scale composed of three items that tapped into the frequency of muscle pain, back pain, and neck pain, and one item that measured the frequency of sleep problems in the last three months. Answers ranged from 1 (*never or almost never*) to 5 (*almost every day or every day*). Internal consistency of the musculoskeletal problems scale was high and stable over time ($\alpha_{January}$ = .79, α_{March} = .75, α_{June} = .75).

Statistical analysis

Preliminary analyses consisted of independent samples t-tests to examine baseline differences in the studied dimensions of well-being between the control condition and intervention condition participants, and between completers and non-completers. This was done to verify that

the effect of the condition was not confounded with the baseline levels of the relevant dimensions of well-being and to check for attrition bias.

The main analyses were latent growth curve models (LGM). LGM is a structural equation modeling approach that models how individuals change over time and how individuals differ from each other in these changes [60]. To reach insight into intraindividual growth and inter-individual differences in growth, LGM introduces two latent growth parameters: an intercept (i.e., baseline level) and a slope (i.e., change over time). Once these latent growth parameters are modeled, covariates can be included to predict them (i.e., to explain why individuals differ in their baseline level and/or change over time). Two types of LGM were conducted in this study: first-order LGM, which investigates change in a single indicator measurement of an observed variable (i.e., concentration problems and sleep problems), and second-order LGM, which is based on a multiple indicator measurement of a latent outcome (i.e., perceived stress, emotional exhaustion, affect, and musculoskeletal problems).

For the latter multi-indicator variables, before performing the second-order LGMs, measurement invariance was established through the testing of configural, metric, and scalar invariance. Configural invariance (i.e., the factor structure is invariant over time), metric invariance (i.e., factor loadings are invariant over time), and scalar invariance (i.e., item intercepts are invariant over time) are required for modeling the latent outcomes in a way that is equivalent over time [61].

Also pertaining to the multi-indicator measures, including design-driven correlated residuals in a model is crucial, as omitting them may cause model misspecification and may change the meaning of and relation between factors [62]. Therefore, residual covariances between the time-specific instances of the same indicator (e.g., indicator 1 at baseline, half-time, and post-intervention) were allowed over the three measurement occasions.

As the central part of the models, the LGMs were performed with a condition dummy (i.e., 0 = control condition versus 1 = intervention condition) as the covariate of the slope factor to test whether the conditions significantly differed in how the well-being dimensions changed over time (i.e., a positive effect of the condition dummy on the slope factor would be indicative of more positive growth in the outcome under study).

Model fit is tapped using several indices, evaluating fit as satisfactory when the following criteria were met: $RMSEA < .06$, $SRMR < .08$, CFI and $TLI > 0.95$, χ^2 $p > .05$ [63–65]. For each LGM (with each LGM corresponding to one outcome), we report the mean estimates for the slope factor in the control condition and the intervention condition, and the difference between those, which corresponds to the regression weight linking the slope factor to the condition dummy. This will allow us to test hypothesis 1. Control condition slope estimates show the predicted change over time (increase when positive or decrease when negative) in the control condition for that dimension of well-being. Intervention condition slope estimates do the same for the intervention condition. The estimated regression weight linking the slope factor to the condition shows the difference between the slopes in the control condition and the intervention condition and thus tests the effect of the intervention. We conducted LGM analyses with 1,000 bootstrap samples in Mplus Version 7 using full information maximum likelihood estimation (i.e., FIML). This type of estimation is recommended to handle missing data as it uses all available information in the data matrix [66].

Afterward, to test hypothesis 2, we used variance decomposition to estimate the proportion of the total variation that can be attributed to the intervention [67]. This technique isolates the proportion of the variance in the well-being measure at post-intervention that is explained by the intervention effect.

To test hypotheses 3a, 3b, and 3c, we tested different functional forms by comparing the default linear LGMs with alternative models involving nonlinear growth terms: quadratic

growth, which would indicate a larger change in outcome in the second half of the study than in the first half, and plateau growth, which would indicate a larger change in outcome in the first half than in the second half of the study. Model fit indices were used to investigate whether any of the nonlinear LGMs would fit the data better than the linear LGMs.

Depending on the well-being outcome, sample sizes used in the analyses varied between $n = 235$ (concentration problems) and $n = 237$ (perceived stress and emotional exhaustion). A full overview of sample size by condition and measurement occasion per dimension of well-being is given in S1 Table.

Results

Descriptive statistics

Table 2 reports the means, standard deviations, and intercorrelations between the studied dimensions of well-being.

The independent samples t-tests showed no significant differences in baseline well-being, neither between the control condition and intervention condition participants nor between the teachers who later dropped out of the study (non-completers) and those who continued to participate (completers) (all $p > .05$). This is true for all dimensions of well-being, except for sleep problems: control condition participants reported more sleep problems at baseline ($p = .019$). S2 Table contains a full report of the t-test results.

Hypotheses testing

Before conducting the LGMs, measurement invariance was established for all dimensions of well-being that were measured by multiple items. Model fit indices showed a well-fitting measurement invariant model for each of the observed dimensions of well-being (lowest *BIC* for

Table 2. Descriptive statistics and correlations between dimensions of well-being.

Well-being measure	M (SD)	1	2	3	4	5	6	7
Emotional Well-Being								
1.Perceived stress	2.68 (0.67)	—						
2.Emotional exhaustion	2.66 (0.85)	.60**	—					
3.Negative affect	2.50 (0.65)	.70***	.54***	—				
4.Positive affect	3.43 (0.57)	-.54***	-.48***	-.45***	—			
Cognitive Well-Being								
5.Concentration problems	2.74 (1.38)	.44***	.44***	-.29***	.46***	—		
Physical Well-Being								
6.Musculoskeletal problems	2.71 (1.19)	.28***	.33***	-.20***	.31***	.31***	—	
7.Sleep problems	2.64 (1.38)	.28***	.28***	-.19***	.28***	.45***	.38***	—

Note. Correlations, means, and standard deviations were computed in the long data format. The correlations of the variables per measurement occasion will be provided on request.

** p < .01

*** p < .001.

https://doi.org/10.1371/journal.pone.0292406.t002

Table 3. Model fit indices for the linear, quadratic, and plateau latent growth models.

Well-being measure	BIC	RMSEA	CFI	TLI	SRMR	χ^2(df)	$\Delta\chi^2$(Δdf)
			Emotional Well-Being				
Perceived stress							
Linear model	**1857.48**	.02	.98	.97	.07	36.94(33)$^{n.s.}$	
Quadratic model	1860.35	.02	.99	.98	.07	34.35(32)$^{n.s.}$	2.59(1)$^{n.s.}$
Plateau model	1866.67	.03	.95	.93	.07	40.66(32)$^{n.s.}$	3.72(1)$^{n.s.}$
Emotional exhaustion							
Linear model	**3003.23**	.04	.99	.99	.06	43.83(33)$^{n.s.}$	
Quadratic model	3010.02	.04	.99	.98	.07	45.16(32)$^{n.s.}$	1.33(1)$^{n.s.}$
Plateau model	3010.63	.04	.99	.98	.07	45.76(32)$^{n.s.}$	1.93(1)$^{n.s.}$
Negative affect							
Linear model	**2568.97**	.03	.99	.99	.06	38.04(33)$^{n.s.}$	
Quadratic model	2575.75	.03	.99	.99	.06	39.35(32)$^{n.s.}$	1.31(1)$^{n.s}$
Plateau model	2576.39	.03	.99	.98	.06	39.99(32)$^{n.s.}$	1.95(1)$^{n.s}$
Positive affect							
Linear model	**2184.26**	.03	.99	.99	.08	38.90(33)$^{n.s.}$	
Quadratic model	2191.52	.03	.99	.98	.08	40.70(32)$^{n.s.}$	1.80(1)$^{n.s.}$
Plateau model	2190.90	.03	.99	.99	.08	40.07(32)$^{n.s}$	1.17(1)$^{n.s.}$
			Cognitive Well-Being				
Concentration problems							
Linear model	**1540.24**	.02	1.00	.99	.04	4.52(4)$^{n.s}$	
Quadratic model	1545.96	.05	.99	.97	.03	4.78(3)$^{n.s}$	0.26(1)$^{n.s}$
Plateau model	1545.94	.05	.99	.97	.04	4.76(3)$^{n.s}$	0.24(1)$^{n.s}$
			Physical Well-Being				
Musculoskeletal problems							
Linear model	**4460.31**	.05	.98	.97	.06	49.55(33)*	
Quadratic model	4465.83	.05	.98	.97	.06	49.60(32)*	0.05(1)$^{n.s}$
Plateau model	4466.31	.06	.97	.96	.06	52.54(32)*	2.99(1)$^{n.s}$
Sleep problems							
Linear model	**1541.02**	.00	1.00	1.03	.01	0.76(2)$^{n.s.}$	
Quadratic model	1545.83	.00	1.00	1.04	.01	0.11(1)$^{n.s.}$	0.65(1)$^{n.s.}$
Plateau model	1546.97	.03	1.00	.99	.03	1.25(1)$^{n.s.}$	0.49(1)$^{n.s.}$

Note. BIC = Bayesian Information Criterion (for each outcome, the lowest BIC model is printed in boldface to mark the preferred model); RMSEA = Root Mean Square Error of Approximation; CFI = comparative fit index; TLI = Tucker-Lewis Index; SRMR = Standardized Root Mean Square Residual; $\Delta\chi^2$(Δdf): chi^2 difference test comparing the linear model to the nonlinear model.

* $p < .05$; n.s. = not significant.

https://doi.org/10.1371/journal.pone.0292406.t003

the scalar invariant model, *RMSEA* < .06, *SRMR* < .08, *CFI* and *TLI* > 0.95, and χ^2 $p > .05$; e.g., [63–65,68]. S3 Table contains a full report of the model fit for the configural, metric, and scalar invariant models for each of the well-being dimensions measured by multiple items.

Table 3 (rows marked with 'linear model') shows the model fit for the linear LGMs, which is satisfactory for each of the well-being dimensions.

Hypothesis 1 states that the focused attention meditation intervention will beneficially affect emotional, cognitive, and physical well-being. This hypothesis can be tested by comparing the intervention condition slopes and the control condition slopes. Fig 1 displays the linear LGM-implied condition trajectories for each of the dimensions of well-being. The findings reported in Table 4 show that during the six-month observation period, control condition

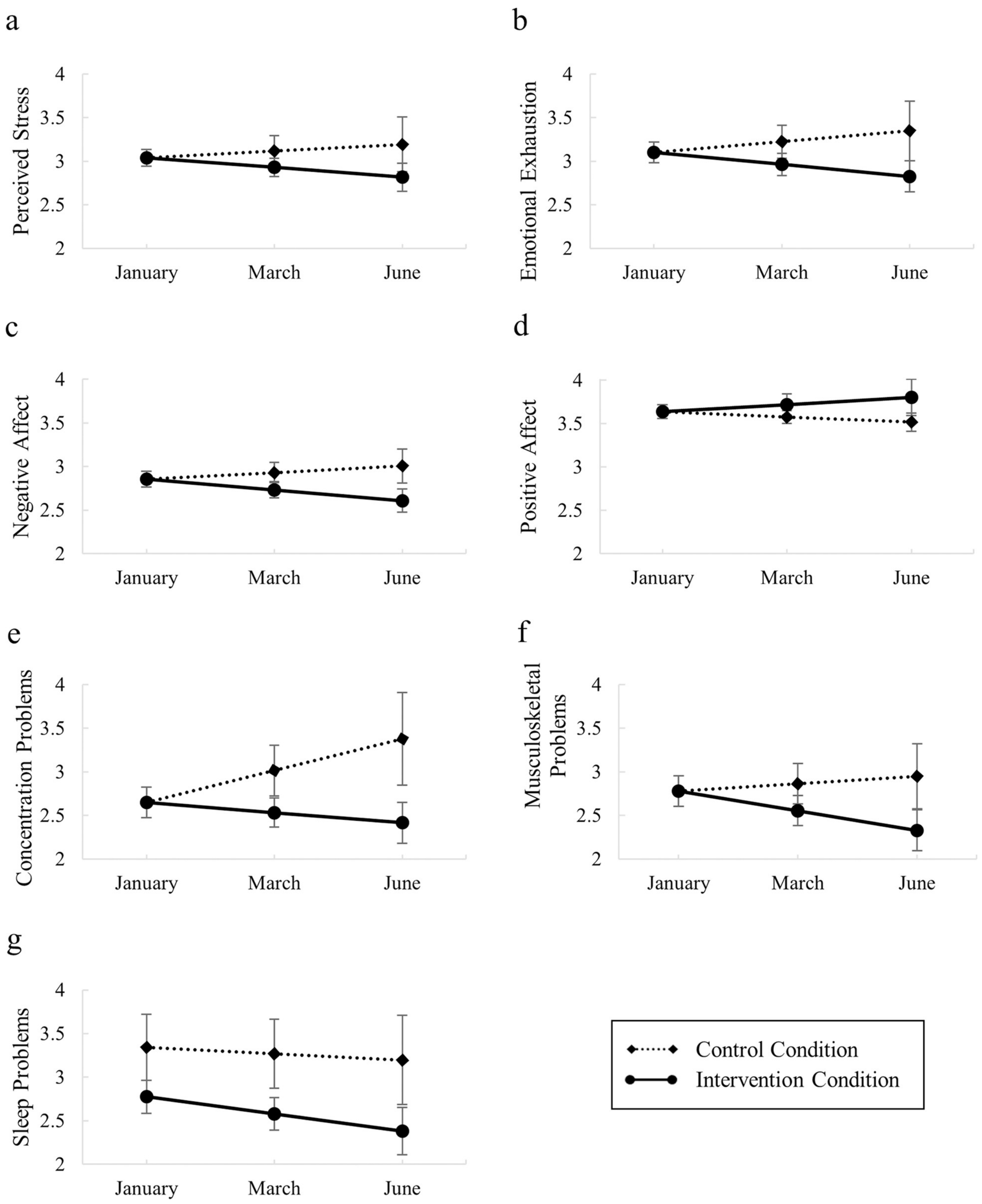

Fig 1. Model implied means and 95% confidence intervals per condition per measurement occasion for (a) perceived stress, (b) emotional exhaustion, (c) negative affect, (d) positive affect, (e) concentration problems, (f) musculoskeletal problems, and (g) sleep problems.

https://doi.org/10.1371/journal.pone.0292406.g001

participants experienced stability in all dimensions of well-being (i.e., the mean slope in the control condition was not statistically significantly different from zero) except for concentration problems, where we observed a significant increase within the control condition. Contrarily, participants who took to practicing focused attention meditation showed significant improvements in all dimensions of well-being (i.e., the mean slope for the intervention condition was statistically significantly different from zero) except for concentration problems, where the improvement was marginally significant (p = .063). Regressing the slope parameter on the condition dummy resulted in significant intervention effects for most of the well-being dimensions (i.e., the slope for the intervention effect was statistically significantly different from zero). The only exception is sleep problems, where the intercept shows that the baseline level was statistically significantly different in the two conditions, a difference that was already established by the independent samples t-test (see S2 Table). Control condition participants did not report a significant change in sleep problems, while participants in the intervention condition experienced a significant decrease. Nonetheless, the test for the intervention effect did not reach statistical significance.

We conclude that hypothesis 1 is supported for the emotional and cognitive well-being indicators and partially supported for the physical well-being indicators.

Hypothesis 2 focused on the effect sizes for each of the dimensions of well-being and predicted a stronger effect size for emotional well-being than for cognitive or physical well-being. To test this, we looked into the amount of variance in the final measurement that can be explained by the intervention, which is also reported in Table 4. The strongest effects of focused attention meditation are found on perceived stress, followed by concentration problems, emotional exhaustion, negative affect, positive affect, and musculoskeletal problems. The explained variances for these outcomes are modest in magnitude and range from 4% to 8%. The effect size for sleep problems was smaller (0.7% explained variance) and not interpretable due to the statistically significant baseline difference between the control condition and the intervention condition. Hypothesis 2 is partially supported: the effect sizes for the emotional well-being indicators are consistently larger than the effect sizes for the physical well-being indicators, but not for the cognitive well-being indicator.

To test hypotheses 3a, 3b, and 3c, the fit of the linear LGMs was compared with the fit of quadratic and plateau LGMs that included either a quadratic or a plateau growth term for each outcome. Table 3 shows that neither of the nonlinear growth models significantly improved the model fit for any of the dimensions of well-being (not significant chi^2 difference test). Additionally, the Bayesian Information Criterion (i.e., BIC) pointed to the linear growth model as being of superior fit in all cases. Taking these comparisons into account, the linear model seemed to reflect the data most adequately. Therefore, we conclude that the effect of the intervention remained constant for all studied dimensions of well-being over the course of six months. These results support hypothesis 3a.

Discussion

Main findings

This study aimed to examine the effect of a focused attention meditation intervention on the emotional, cognitive, and physical well-being of elementary school teachers over the course of six months during the COVID-19 pandemic. The intervention entailed a few minutes of classroom meditation with the pupils at the beginning of each school day. Teachers and pupils were encouraged, but not obliged, to engage in additional meditation practice during non-school hours. The meditation intervention was set up as a free program in which teachers

Table 4. Estimates for second-order latent growth model: Coefficients, significance levels, standard errors, effect sizes.

Parameter	Estimate	SE	R^2
Emotional Well-Being			
Perceived stress (*n* = 237)			
Control condition slope	.08$^{n.s.}$	0.08	
Intervention condition slope	-.11**	0.04	
Intervention effect	-.19*	0.10	.08
Emotional exhaustion (*n* = 237)			
Control condition slope	.12$^{n.s.}$	0.09	
Intervention condition slope	-.14**	0.04	
Intervention effect	-.26**	0.09	.05
Negative affect (*n* = 236)			
Control condition slope	.08$^{n.s.}$	0.05	
Intervention condition slope	-.12***	0.03	
Intervention effect	-.20**	0.06	.05
Positive affect (*n* = 236)			
Control condition slope	-.06$^{n.s.}$	0.05	
Intervention condition slope	.08**	0.03	
Intervention effect	.14*	0.06	.04
Cognitive Well-Being			
Concentration problems (*n* = 235)			
Control condition slope	.37**	0.14	
Intervention condition slope	-.12†	0.06	
Intervention effect	-.48**	0.15	.07
Physical Well-Being			
Musculoskeletal problems (*n* = 236)			
Control condition slope	.09$^{n.s.}$	0.09	
Intervention condition slope	-.23***	0.06	
Intervention effect	-.31**	0.11	.04
Sleep problems (*n* = 236)			
Intercept difference	-.57**	0.22	
Control condition slope	-.07$^{n.s.}$	0.11	
Intervention condition slope	-.20**	0.07	
Intervention effect	-.13$^{n.s.}$	0.13	.007

Note. Intervention effect = condition (0 = control condition, 1 = intervention condition) as a dichotomous predictor of the linear slope; Intercept difference = condition as a dichotomous predictor of baseline well-being; R^2 = proportion of total variance explained by intervention.

† $p < .10$

* $p < .05$

** $p < .01$

*** $p < .001$; n.s. = not significant.

https://doi.org/10.1371/journal.pone.0292406.t004

could voluntarily engage to deal with the exacerbated levels of stress and strain that COVID-19 brought about.

Multidimensional well-being. Our findings show a consistent beneficial effect of focused attention meditation on emotional, cognitive, and physical well-being in participating teachers during our six-month observation window. These findings align with research findings in pre-

COVID times that tap into the effect of focused attention meditation and mindfulness-based interventions on well-being [19,31,33].

However, contrary to previous research, our findings suggest that the well-being benefits of meditation might manifest themselves via two different causal pathways. The first pathway is suggested in most former meditation studies and states that meditation practice leads to increased levels of well-being in the intervention condition. In our study, this pathway was observed for perceived stress, emotional exhaustion, negative affect, positive affect, and musculoskeletal problems. No changes were observed in the control condition. A similar observation was done for sleep problems, but the intervention effect was not statistically significant at $p < .05$-level. The second pathway shows that meditation practice does not necessarily lead to increases in levels of well-being, but that it prevents levels of well-being from decreasing in case of exacerbated levels of stress and strain. In our study, we observed this pathway for concentration problems. Only a limited number of studies have reported this prophylactic effect for mindfulness-based interventions [32,69,70] and focused attention meditation [69].

Differential effects on well-being dimensions. Our results point out that focused attention meditation has the strongest effect on emotional and cognitive well-being, while the effect on physical well-being was smaller. The finding that the effect was larger for emotional well-being than for physical well-being aligns with previous research on mindfulness-based interventions [44]. When it comes to cognitive well-being, the knowledge base was more limited. Some mindfulness studies reported small effect sizes [45,46], but the centrality of attention in the focused attention meditation technique made somewhat larger effect sizes plausible for this study.

Changes in well-being effects over time. We tested a linear model, a quadratic model, and a plateauing model and found evidence supporting a linear change pattern. Our findings cannot exclude that the few minutes of daily focused attention meditation may have been insufficient to trigger an acceleration effect. Previous studies that observed a quadratic pattern [49,51] focused on more intensive mindfulness-based interventions with longer daily meditation sessions. The possibility of a plateau effect has not yet been empirically tested elsewhere [50]. It remains unclear whether meditation practice is subdued to a mechanism of diminishing returns.

Theoretical contributions

This study adds to the literature on occupational well-being in three major ways.

First, it expands our understanding of focused attention meditation as an intervention technique in stress management programs addressing occupational well-being. Mindfulness-based interventions have received a great deal of attention, but these interventions usually encompass a variety of techniques, disabling to pinpoint the well-being effects of specific constituting parts. Our study focused on one such part: focused attention meditation. Focused attention meditation is part of most mindfulness-based interventions as it is considered to be an accessible starting point for meditation novices.

Second, our multidimensional perspective on well-being enables us to assess the effect of focused attention meditation on emotional, cognitive, and physical well-being, and to compare the relative strengths of the effects. No prior studies on focused attention meditation or mindfulness-based interventions have simultaneously addressed these three dimensions of well-being. In workplace studies on mindfulness-based interventions, physical well-being has been largely understudied [30].

Third, our study covers a six-month time window and encompasses three points of measurement, while most studies investigating mindfulness-based intervention effects employ a

pre- and post-measurement in a six to eight-week time frame [33,71]. Our approach allowed us to monitor changes in effects over a longer period and to test different change patterns. Existing meditation research on this issue is scarce and provides empirical results on two of the three change patterns (i.e., linear growth and quadratic growth, see [49–51]), but only for mindfulness-based interventions. This is the first workplace meditation study that has empirically tested for the plateau pattern.

Limitations and future directions

A few limitations of this study should be kept in mind.

One limitation, common in longitudinal research [72], is that our study was prone to attrition. Two consequences are worth mentioning. The first is that attrition bias might impair the interpretation of our results. Such bias emerges when a drop in the level of well-being is accompanied by a higher drop-out rate. The current study focused on teachers, an occupational group that is burdened by high rates of stress and work overload and that is prone to high rates of burn-out all over the world [28,73]. While we cannot entirely exclude such bias, attrition analyses of our data showed that levels of well-being in a prior survey round of our study could not predict attrition in a later survey round. In case a non-observed attrition bias would exist, the intervention effects might be overestimated. A second consequence of attrition in longitudinal studies is that effective sample sizes decrease, hampering fine-grained analyses. In our study, effective sample sizes did not allow us to disentangle the effects of focused attention meditation on the well-being of those teachers who had prior experience with meditation and those who did not. Similarly, we cannot compare teachers who engaged in extra meditation practice at home with those restricting their meditation practice to the daily sessions in the classroom. Further research might tap into this issue, as we might hypothesize that the well-being benefits of classroom meditation in teachers might not only be triggered directly by their meditation practice but also indirectly by meditation-induced changes in classroom dynamics (e.g., improved relationship quality among pupils, and increased pupil well-being). Additionally, the frequency and duration of the at-home meditation practice may have influenced the magnitude of the well-being effects. Although the scientific community has not reached a consensus regarding the 'optimal' frequency and duration of meditation sessions yet [74], a 2017 systematic review found indications that practising meditation more often could lead to greater well-being benefits [75]. However, the systematic review does not focus on workplace interventions and the sample size in the current study unfortunately did not allow us to investigate this moderation. Future research is needed to build a scientific consensus on the frequency and duration of meditation sessions that is needed to create optimal intervention effectiveness.

A second limitation to consider is that our sample is based on the self-selection of respondents. Janssen and colleagues [30] have stated that this is a general issue in meditation research since most studies employ samples of people who volunteered to participate. Self-selection might have several implications. One implication is that meditating participants might differ significantly from the control condition on an important non-measured determinant of occupational well-being. While we can never completely rule this out, analyses of the measured determinants of occupational well-being (analyses not shown) indicated no significant differences at baseline. Another implication of self-selection is that those participants voluntarily engaging in a well-being intervention might be more aware of their well-being and the changes therein. Additionally, participants who voluntarily engage in an intervention might be especially susceptible to the beneficial effects that the intervention might bring [76] or they might experience increases in well-being due to the mere fact of participation and expectation. The

use of a waitlist control condition in our study cannot entirely rule out the possibility of such a placebo effect. Future research might test for placebo by comparing a focused attention meditation condition to an active control condition (e.g., other meditation techniques, yoga, physical activity). A final implication of self-selection worth mentioning is that it might put restrictions on the generalizability of findings. However, a comparison of participating teachers and schools in our study with official statistics on education in Flanders shows that the study participants accurately reflected the regional teacher population in terms of gender, age, and employment contract. Additionally, the participating schools accurately reflected the wide range of institutional, organizational, and religious contexts that characterize the Flemish elementary education sector. This observation suggests high levels of generalizability of the meditation intervention. This generalizability might expand to other professions and sectors of the labor market. Nonetheless, the fact that the vast majority of participants in this sample was female, warrants extra caution when generalizing these finding to male employees. Previous research in a sample of college students indicated the possibility that well-being effects of meditation may be larger for female college students than for their male counterparts [77]. Future research might benefit from samples with a more balanced gender distribution, so that gender differences in the well-being effects of workplace meditation interventions can be investigated.

A potential third limitation relates to the fact that the intervention and study took place during the COVID-19 pandemic. During the study, the federal and regional governments imposed strict rules on social interactions and closed down all workplaces that were considered 'non-essential'. Elementary schools were kept open at all times and teachers were supposed to continue their normal teaching but also to protect themselves by wearing a face mask and sticking to strict hygienic rules. Pupils did not wear face masks and returned home to their families at the end of each school day. This resulted in an increased risk of COVID-infection for teachers. As the government rapidly responded to the waves of the pandemic by imposing and relaxing restrictions, working conditions changed repeatedly, adding to heavy workloads and heightened and chronic stress that the teaching population continuously reported before COVID-19 [3,73,78]. Are the well-being benefits of focused attention meditation different in periods of heightened chronic levels of stress? In case this is a fact, some bias might occur.

A fourth limitation of our study is that it cannot pinpoint why and how the effects of focused attention meditation came about. Future research might benefit from exploring the interplay of well-being outcomes and dimensions over time, disentangling triggering, accelerating, and mediating dynamics. In addition, future research might also benefit from addressing the underlying biological, psychological, and social mechanisms that might be at play when focused attention meditation becomes a factor in the stress management process. Research into self-determination, emotion, but also compassion, tolerance, and team performance might be especially fruitful.

Finally, this study did not consider the potential effects of meditation on spiritual well-being. While meditation originated in Buddhist traditions, it can be practiced by people of all religious and non-religious backgrounds. Meditation might help one to find meaning in life and work, a possibility that was not explored in this study. Future research might investigate this issue further by including measures of spiritual well-being such as the Spiritual Well-Being Scale developed by Paloutzian and Ellison [79]. This scale is non-sectarian and can thus be used in a wide range of settings, for participants from different backgrounds and with different beliefs.

Practical implications

Organizations spend considerable resources on stress management programs to optimize occupational well-being and several interventions have been shown to be effective [80].

Mindfulness-based interventions have witnessed increased popularity both outside and inside the workplace. These interventions vary greatly in duration and intensity, with some programs requiring daily meditation sessions of at least 15 minutes, either individually or in a group [33] and other programs entailing intense practice schedules, with weekly two-and-a-half hour group sessions, daily home practices of 30 minutes, and a silent retreat of four to eight hours [81]. Focused attention meditation is a core part of many of these interventions and is considered to be low threshold for novices [23]. In the current study, findings show that a few minutes of daily focused attention meditation at work is an effective intervention to address employees' emotional, cognitive, and physical well-being and that it may even offset the health impairing effects of times of crisis, such as the COVID-19 pandemic. This effectiveness comes with some additional advantages compared to many other well-being interventions.

First, the financial costs of initiating and continuing focused attention meditation in the workplace are limited compared to many other interventions, as they do not require specific equipment, additional workspace, intensive teaching, or regular follow-up by a paid trainer or instructor. The present study demonstrates that significant occupational well-being benefits can be triggered by a few minutes of daily focused attention meditation as taught in a single, group-based online training session.

Second, the practice of focused attention meditation is in essence time and place independent. It can be practiced at all times of the day, enabling practitioners to combine it with flexible and changing work schedules. It can be practiced in and outside the office or workplace, which is a major advantage in times when teleworking and hybrid work formats are on the rise, and many businesses close down or downsize their physical offices.

Third, focused attention meditation is an intervention that can be practiced individually or in a team. Managers and team leaders can consider meditation as a tool when addressing team dynamics and team building. Research on mindfulness-based interventions observed increases in team performance and cohesion when each team member engaged in the meditation individually [82] or as a team [83]. However, it should be kept in mind that our findings are based on a focused attention meditation intervention in which participation was voluntary, and making meditation practice obligatory in the workplace might not trigger similar well-being benefits as observed in our study. Activities performed out of autonomous motivation are more durable, and compliance with meditation practices may be higher among autonomously motivated employees [84]. However, autonomous motivation to meditate might be triggered through communication about possible advantages, providing adequate support (e.g., training platforms, quiet spaces), or encouraging brief meditation sessions during work time [85].

Fourth, focused attention meditation is compatible with all kinds of change programs in the structure and culture of organizations. This compatibility is important as meditation cannot make up for dysfunctional teams or companies. Meditation may be perceived as a personal resource that helps to build employee resilience [86–88], but it cannot neutralize problematic work contexts.

Conclusions

The current study assessed the effect of a focused attention meditation intervention on occupational well-being during COVID-19 times. Findings show that in the course of six months, a few minutes of meditation a day triggered significant benefits for emotional, cognitive, and physical well-being. Meditation practice led to well-being benefits either by promoting well-being (in the case of emotional and physical well-being) or by protecting it from worsening (in the case of cognitive well-being).

Supporting information

S1 Table. Sample Size (n) by condition and time per dimension of well-being.
(DOCX)

S2 Table. Well-being at baseline: Independent samples t-test based on condition (0 = control condition, 1 = intervention condition) and based on completion (0 = noncomplete, 1 = complete).
(DOCX)

S3 Table. Model fit indices for the configural invariant, metric invariant, and scalar invariant models.
(DOCX)

Author Contributions

Conceptualization: Justine Van de Velde, Katia Levecque, Steven Laureys.

Data curation: Justine Van de Velde, Katia Levecque.

Formal analysis: Justine Van de Velde, Bert Weijters.

Funding acquisition: Katia Levecque.

Investigation: Justine Van de Velde, Katia Levecque.

Methodology: Katia Levecque, Bert Weijters.

Project administration: Justine Van de Velde, Katia Levecque.

Resources: Katia Levecque.

Supervision: Katia Levecque.

Validation: Justine Van de Velde, Katia Levecque.

Visualization: Justine Van de Velde, Bert Weijters.

Writing – original draft: Justine Van de Velde, Katia Levecque.

Writing – review & editing: Justine Van de Velde, Katia Levecque, Bert Weijters, Steven Laureys.

References

1. World Health Organization. Mental health and COVID-19: Early evidence of the pandemic's impact. Scientific brief. 2022. Available: https://www.who.int/publications/i/item/WHO-2019-nCoV-Sci_Brief-Mental_health-2022.1.
2. De Laet H, Verhavert Y, De Martelaer K, Zinzen E, Deliens T, Van Hoof E. Impact of the COVID-19 pandemic on risk of burn-out syndrome and recovery need among secondary school teachers in Flanders: A prospective study. Front Public Health. 2022;10. https://doi.org/10.3389/fpubh.2022.1046435 PMID: 36579060
3. Hutchison SM, Watts A, Gadermann A, Oberle E, Oberlander TF, Lavoie PM, et al. School staff and teachers during the second year of COVID-19: Higher anxiety symptoms, higher psychological distress, and poorer mental health compared to the general population. J Affect Disord Rep. 2022;8. https://doi.org/10.1016/j.jadr.2022.100335 PMID: 35313684
4. Young KP, Kolcz DL, O'Sullivan DM, Ferrand J, Fried J, Robinson K. Health Care Workers' Mental Health and Quality of Life During COVID-19: Results From a Mid-Pandemic, National Survey. Psychiatric Services. 2021; 72: 122–128. https://doi.org/10.1176/appi.ps.202000424 PMID: 33267652
5. American Federation of Teachers. 2017 Educator Quality of Work Life Survey. 2017. Available: https://www.aft.org/sites/default/files/media/2017/2017_eqwl_survey_web.pdf.

6. Eurofound. Working conditions and workers' health. Publications Office of the European Union. 2019. Available: https://www.eurofound.europa.eu/publications/report/2019/working-conditions-and-workers-health#tab-02.
7. Health and Safety Executive. Education statistics in Great Britain 2020. 2020. Available: www.hse.gov.uk/statistics/industry/sic2007.htm.
8. Education Support Partnership. Teacher Wellbeing Index 2018. 2018.
9. United Nations. Coronavirus disease (COVID-19) | Wellness tips. [cited 27 Mar 2023]. Available: https://www.un.org/en/coronavirus/wellness#:~:text=Wellbeing%20tips%20for%20UN%20Personnel&text=Remember%20basic%20well-being%20practices,Practice%20mindfulness.
10. Jamieson SD, Tuckey MR. Mindfulness interventions in the workplace: A critique of the current state of the literature. J Occup Health Psychol. 2017; 22: 180–193. https://doi.org/10.1037/ocp0000048 PMID: 27643606
11. Kabat-Zinn J. Mindfulness-Based Interventions in Context: Past, Present, and Future. Clinical Psychology: Science and Practice. 2003; 10: 144–156. https://doi.org/10.1093/clipsy/bpg016
12. Segal Z V., Williams JMG, Teasdale JD. Mindfulness-based cognitive therapy for depression. New York: The Guilford Press; 2013.
13. Linehan MM. Cognitive Behavioral Treatment of borderline personality disorder. New York: The Guilford Press; 1993.
14. Hayes SC, Luoma JB, Bond FW, Masuda A, Lillis J. Acceptance and Commitment Therapy: Model, processes and outcomes. Behaviour Research and Therapy. 2006; 44: 1–25. https://doi.org/10.1016/j.brat.2005.06.006 PMID: 16300724
15. Keng S-L, Smoski MJ, Robins CJ. Effects of mindfulness on psychological health: A review of empirical studies. Clin Psychol Rev. 2011; 31: 1041–1056. https://doi.org/10.1016/j.cpr.2011.04.006 PMID: 21802619
16. Lazarus RS, Folkman S. Stress, Appraisal, and Coping. New York, NY: Springer Publishing Company; 1984.
17. Gomes AR, Faria S, Gonçalves AM. Cognitive appraisal as a mediator in the relationship between stress and burnout. Work Stress. 2013; 27: 351–367.
18. Gardner B, Rose J, Mason O, Tyler P, Cushway D. Cognitive therapy and behavioural coping in the management of work-related stress: An intervention study. Work Stress. 2005; 19: 137–152. https://doi.org/10.1080/02678370500157346
19. Laureys S. The no-nonsense meditation book: A scientist's guide to the power of meditation. Green Tree; 2021.
20. Benson H, Klipper MZ. The Relaxation Response. HarperCollins; 2009.
21. Nixon AE, Mazzola JJ, Bauer J, Krueger JR, Spector PE. Can work make you sick? A meta-analysis of the relationships between job stressors and physical symptoms. Work Stress. 2011; 25: 1–22. https://doi.org/10.1080/02678373.2011.569175
22. Kemper KJ, Rao N. Brief Online Focused Attention Meditation Training: Immediate Impact. J Evid Based Complementary Altern Med. 2017; 22: 395–400. https://doi.org/10.1177/2156587216663565 PMID: 27530315
23. Lohani M, McElvaine K, Payne B, Mitcheom K, Britton W. A Longitudinal Training Study to Delineate the Specific Causal Effects of Open Monitoring Versus Focused Attention Techniques on Emotional Health. Complement Ther Med. 2020; 53: 102525. https://doi.org/10.1016/j.ctim.2020.102525 PMID: 33066868
24. Lutz A, Slagter HA, Dunne JD, Davidson RJ. Attention regulation and monitoring in meditation. Trends Cogn Sci. 2008; 12: 163–169. https://doi.org/10.1016/j.tics.2008.01.005 PMID: 18329323
25. Margolis S, Schwitzgebel E, Ozer DJ, Lyubomirsky S. Empirical Relationships Among Five Types of Well-Being. In: Lee MT, Kubzansky LD, VanderWeele TJ, editors. Measuring Well-Being. Oxford University Press; 2021. https://doi.org/10.1093/oso/9780197512531.003.0014
26. Joshanloo M, Weijers D. A two-dimensional conceptual framework for understanding mental well-being. Thrash T, editor. PLoS One. 2019;14. https://doi.org/10.1371/journal.pone.0214045 PMID: 30917191
27. Halbreich U. Stress-related physical and mental disorders: a new paradigm. BJPsych Adv. 2021; 27: 145–152. https://doi.org/10.1192/bja.2021.1
28. Bourdeaud'hui R, Janssens F, Vanderhaeghe S. Vlaamse werkbaarheidsmonitor 2019—Werknemers. Brussel; 2019. Available: https://www.serv.be/sites/default/files/documenten/StIA_20191130_WerkbaarheidsmonitorWerknemers2019_RAP.pdf.

29. Carstarphen M. Putting Out Teacher Burnout. 2022. Available: https://news.gallup.com/opinion/gallup/400670/putting-teacher-burnout.aspx.

30. Janssen M, Heerkens Y, Kuijer W, van der Heijden B, Engels J. Effects of Mindfulness-Based Stress Reduction on employees' mental health: A systematic review. Ebmeier K, editor. PLoS One. 2018; 13: e0191332. https://doi.org/10.1371/journal.pone.0191332 PMID: 29364935

31. Zarate K, Maggin DM, Passmore A. Meta-analysis of mindfulness training on teacher well-being. Psychol Sch. 2019; 56: 1700–1715. https://doi.org/10.1002/pits.22308

32. Jha AP, Stanley EA, Kiyonaga A, Wong L, Gelfand L. Examining the protective effects of mindfulness training on working memory capacity and affective experience. Emotion. 2010; 10: 54–64. https://doi.org/10.1037/a0018438 PMID: 20141302

33. Pérez-Fuentes M del C, Molero Jurado M del M, Mercader Rubio I,Soriano Sánchez JGGázquez Linares JJ. Mindfulness for Preventing Psychosocial Risks in the Workplace: A Systematic Review and Meta-Analysis. Applied Sciences. 2020; 10: 1851. https://doi.org/10.3390/app10051851

34. Jennings PA, Frank JL, Snowberg KE, Coccia MA, Greenberg MT. Improving classroom learning environments by Cultivating Awareness and Resilience in Education (CARE): Results of a randomized controlled trial. School Psychology Quarterly. 2013; 28: 374–390. https://doi.org/10.1037/spq0000035 PMID: 24015983

35. Taylor C, Harrison J, Haimovitz K, Oberle E, Thomson K, Schonert-Reichl K, et al. Examining Ways That a Mindfulness-Based Intervention Reduces Stress in Public School Teachers: a Mixed-Methods Study. Mindfulness (N Y). 2016; 7: 115–129. https://doi.org/10.1007/s12671-015-0425-4

36. Viac C, Fraser P. Teachers' well-being: A framework for data collection and analysis. OECD Education Working Papers. 2020 [cited 3 Dec 2020]. https://doi.org/10.1787/c36fc9d3-en

37. Travis F, Shear J. Focused attention, open monitoring and automatic self-transcending: Categories to organize meditations from Vedic, Buddhist and Chinese traditions. Conscious Cogn. 2010; 19: 1110–1118. https://doi.org/10.1016/j.concog.2010.01.007 PMID: 20167507

38. Verhoeven C, Maes S, Kraaij V, Joekes K. The Job Demand-Control-Social Support Model and Wellness/Health Outcomes: A European Study. Psychol Health. 2003; 18: 421–440. https://doi.org/10.1080/0887044031000147175

39. Bogaert I, De Martelaer K, Deforche B, Clarys P, Zinzen E. Associations between different types of physical activity and teachers' perceived mental, physical, and work-related health. BMC Public Health. 2014; 14: 534. https://doi.org/10.1186/1471-2458-14-534 PMID: 24885620

40. Michalsen A, Kunz N, Jeitler M, Brunnhuber S, Meier L, Lüdtke R, et al. Effectiveness of focused meditation for patients with chronic low back pain—A randomized controlled clinical trial. Complement Ther Med. 2016; 26: 79–84. https://doi.org/10.1016/j.ctim.2016.03.010 PMID: 27261986

41. Jeitler M, Brunnhuber S, Meier L, Lüdtke R, Büssing A, Kessler C, et al. Effectiveness of Jyoti Meditation for Patients With Chronic Neck Pain and Psychological Distress—A Randomized Controlled Clinical Trial. J Pain. 2015; 16: 77–86. https://doi.org/10.1016/j.jpain.2014.10.009 PMID: 25451627

42. Dunne PJ, Lynch J, Prihodova L, O'Leary C, Ghoreyshi A, Basdeo SA, et al. Burnout in the emergency department: Randomized controlled trial of an attention-based training program. J Integr Med. 2019; 17: 173–180. https://doi.org/10.1016/j.joim.2019.03.009 PMID: 30956141

43. Jennings PA, Frank JL, Snowberg KE, Coccia MA, Greenberg MT. Improving classroom learning environments by Cultivating Awareness and Resilience in Education (CARE): Results of a randomized controlled trial. School Psychology Quarterly. 2013; 28: 374–390. https://doi.org/10.1037/spq0000035 PMID: 24015983

44. Goldberg SB, Riordan KM, Sun S, Davidson RJ. The Empirical Status of Mindfulness-Based Interventions: A Systematic Review of 44 Meta-Analyses of Randomized Controlled Trials. Perspectives on Psychological Science. 2022; 17: 108–130. https://doi.org/10.1177/1745691620968771 PMID: 33593124

45. Sumantry D, Stewart KE. Meditation, Mindfulness, and Attention: a Meta-analysis. Mindfulness (N Y). 2021; 12: 1332–1349. https://doi.org/10.1007/s12671-021-01593-w

46. Flook L, Goldberg SB, Pinger L, Bonus K, Davidson RJ. Mindfulness for Teachers: A Pilot Study to Assess Effects on Stress, Burnout, and Teaching Efficacy. Mind, Brain, and Education. 2013; 7: 182–195. https://doi.org/10.1111/mbe.12026 PMID: 24324528

47. Shapiro DH. A preliminary study of long term meditators: Goals, effects, religious orientation, cognitions. Journal of Transpersonal Psychology. 1992; 24: 23–39.

48. Luberto CM, Shinday N, Song R, Philpotts LL, Park ER, Fricchione GL, et al. A Systematic Review and Meta-analysis of the Effects of Meditation on Empathy, Compassion, and Prosocial Behaviors. Mindfulness (N Y). 2018; 9: 708–724. https://doi.org/10.1007/s12671-017-0841-8 PMID: 30100929

49. Labelle LE, Campbell TS, Faris P, Carlson LE. Mediators of Mindfulness-Based Stress Reduction (MBSR): Assessing the Timing and Sequence of Change in Cancer Patients. J Clin Psychol. 2015; 71: 21–40. https://doi.org/10.1002/jclp.22117 PMID: 25099479

50. Hülsheger UR, Feinholdt A, Nübold A. A low-dose mindfulness intervention and recovery from work: Effects on psychological detachment, sleep quality, and sleep duration. J Occup Organ Psychol. 2015; 88: 464–489. https://doi.org/10.1111/joop.12115

51. Wachholtz A, Vohra R, Metzger A. A reanalysis of a randomized trial on meditation for migraine headaches: Distraction is not enough but meditation takes time. Complement Ther Med. 2019; 46: 136–143. https://doi.org/10.1016/j.ctim.2019.08.011 PMID: 31519270

52. Collishaw S. Annual Research Review: Secular trends in child and adolescent mental health. Journal of Child Psychology and Psychiatry. 2015; 56: 370–393. https://doi.org/10.1111/jcpp.12372 PMID: 25496340

53. Bignardi G, Dalmaijer ES, Anwyl-Irvine AL, Smith TA, Siugzdaite R, Uh S, et al. Longitudinal increases in childhood depression symptoms during the COVID-19 lockdown. Arch Dis Child. 2021; 106: 791–797. https://doi.org/10.1136/archdischild-2020-320372 PMID: 33298552

54. Martiny SE, Thorsteinsen K, Parks-Stamm EJ, Olsen M, Kvalø M. Children's Well-being during the COVID-19 pandemic: Relationships with attitudes, family structure, and mothers' Well-being. European Journal of Developmental Psychology. 2022; 19: 711–731. https://doi.org/10.1080/17405629.2021.1948398

55. Cohen S, Kamarck T, Mermelstein R. A Global Measure of Perceived Stress. J Health Soc Behav. 1983; 24: 385. https://doi.org/10.2307/2136404 PMID: 6668417

56. Kokkinos CM. Factor structure and psychometric properties of the Maslach Burnout Inventory-Educators Survey among elementary and secondary school teachers in Cyprus. Stress and Health. 2006; 22: 25–33. https://doi.org/10.1002/smi.1079

57. Maslach C, Jackson SE. Maslach Burnout Inventory manual. 2nd ed. Palo Alto, CA.m: Consulting Psychologists Press; 1986. Available: https://www.researchgate.net/publication/277816643.

58. Watson D, Clark LA, Tellegen A. Development and validation of brief measures of positive and negative affect: The PANAS scales. J Pers Soc Psychol. 1988; 54: 1063–1070. https://doi.org/10.1037//0022-3514.54.6.1063 PMID: 3397865

59. Fisher GG, Matthews RA, Gibbons AM. Developing and investigating the use of single-item measures in organizational research. J Occup Health Psychol. 2016; 21: 3–23. https://doi.org/10.1037/a0039139 PMID: 25894198

60. Muthen BO, Khoo S-T. Longitudinal studies of achievement growth using latent variable modeling. Learn Individ Differ. 1998; 10: 73–101. https://doi.org/10.1016/S1041-6080(99)80135-6

61. Ployhart RE, Vandenberg RJ. Longitudinal Research: The Theory, Design, and Analysis of Change. J Manage. 2010; 36: 94–120. https://doi.org/10.1177/0149206309352110

62. Cole DA, Ciesla JA, Steiger JH. The insidious effects of failing to include design-driven correlated residuals in latent-variable covariance structure analysis. Psychol Methods. 2007; 12: 381–398. https://doi.org/10.1037/1082-989X.12.4.381 PMID: 18179350

63. Hooper D, Coughlan J, Mullen MR. Structural equation modelling: Guidelines for determining model fit. Electronic Journal of Business Research Methods. 2008; 6: 53–60. https://doi.org/10.21427/D7CF7R

64. Byrne BM. Structural equational modeling with AMOS: Basic concepts, applications, and programming. 2nd ed. Harlow L, editor. Taylor & Francis Group; 2010.

65. Hu L, Bentler PM. Cutoff criteria for fit indexes in covariance structure analysis: Conventional criteria versus new alternatives. Struct Equ Modeling. 1999; 6: 1–55. https://doi.org/10.1080/10705519909540118

66. Newman DA. Missing Data. Organ Res Methods. 2014; 17: 372–411. https://doi.org/10.1177/1094428114548590

67. Rights JD, Sterba SK. Quantifying explained variance in multilevel models: An integrative framework for defining R-squared measures. Psychol Methods. 2019; 24: 309–338. https://doi.org/10.1037/met0000184 PMID: 29999378

68. Cheung GW, Rensvold RB. Evaluating Goodness-of-Fit Indexes for Testing Measurement Invariance. Struct Equ Modeling. 2002; 9: 233–255. https://doi.org/10.1207/S15328007SEM0902_5

69. Borchardt AR, Zoccola PM. Recovery from stress: an experimental examination of focused attention meditation in novices. J Behav Med. 2018; 41: 836–849. https://doi.org/10.1007/s10865-018-9932-9 PMID: 29850971

70. Banks JB, Welhaf MS, Srour A. The protective effects of brief mindfulness meditation training. Conscious Cogn. 2015; 33: 277–285. https://doi.org/10.1016/j.concog.2015.01.016 PMID: 25680006

71. Kreplin U, Farias M, Brazil IA. The limited prosocial effects of meditation: A systematic review and meta-analysis. Sci Rep. 2018; 8: 2403. https://doi.org/10.1038/s41598-018-20299-z PMID: 29402955

72. Gustavson K, von Soest T, Karevold E, Røysamb E. Attrition and generalizability in longitudinal studies: findings from a 15-year population-based study and a Monte Carlo simulation study. BMC Public Health. 2012; 12: 918. https://doi.org/10.1186/1471-2458-12-918 PMID: 23107281

73. American Psychological Association. The American workforce faces compounding pressure—APA's 2021 Work and Well-being Survey results. 2021 [cited 12 Oct 2022]. Available: https://www.apa.org/pubs/reports/work-well-being/compounding-pressure-2021.

74. Williams EC, Polito V. Meditation in the Workplace: Does Mindfulness Reduce Bias and Increase Organisational Citizenship Behaviours? Front Psychol. 2022;13. https://doi.org/10.3389/fpsyg.2022.747983 PMID: 35478759

75. Parsons CE, Crane C, Parsons LJ, Fjorback LO, Kuyken W. Home practice in Mindfulness-Based Cognitive Therapy and Mindfulness-Based Stress Reduction: A systematic review and meta-analysis of participants' mindfulness practice and its association with outcomes. Behaviour Research and Therapy. 2017; 95: 29–41. https://doi.org/10.1016/j.brat.2017.05.004 PMID: 28527330

76. Williams AL, Dixon J, McCorkle R, van Ness PH. Determinants of meditation practice inventory: Development, content validation, and initial psychometric testing. Altern Ther Health Med. 2011; 17: 16–23. PMID: 22314672

77. Rojiani R, Santoyo JF, Rahrig H, Roth HD, Britton WB. Women Benefit More Than Men in Response to College-based Meditation Training. Front Psychol. 2017; 8: 1–11. https://doi.org/10.3389/fpsyg.2017.00551 PMID: 28473783

78. Kush JM, Badillo-Goicoechea E, Musci RJ, Stuart EA. Teachers' Mental Health During the COVID-19 Pandemic. Educational Researcher. 2022; 51: 593–597. https://doi.org/10.3102/0013189X221134281

79. Paloutzian RF, Ellison CW. Spiritual Well-Being Scale. 1982. Available: https://www.westmont.edu/psychology/raymond-paloutzian-spiritual-wellbeing-scale.

80. Sakuraya A, Imamura K, Watanabe K, Asai Y, Ando E, Eguchi H, et al. What Kind of Intervention Is Effective for Improving Subjective Well-Being Among Workers? A Systematic Review and Meta-Analysis of Randomized Controlled Trials. Front Psychol. 2020;11. https://doi.org/10.3389/fpsyg.2020.528656 PMID: 33281653

81. Kabat-Zinn J. Full catastrophe living: Using the wisdom of your body and mind to face stress, pain and illness. New York: Delacourt; 1990.

82. Kelm DJ, Ridgeway JL, Gas BL, Mohan M, Cook DA, Nelson DR, et al. Mindfulness Meditation and Interprofessional Cardiopulmonary Resuscitation: A Mixed-Methods Pilot Study. Teach Learn Med. 2018; 30: 433–443. https://doi.org/10.1080/10401334.2018.1462186 PMID: 29775080

83. Colgan DD, Christopher M, Bowen S, Brems C, Hunsinger M, Tucker B, et al. Mindfulness-based Wellness and Resilience intervention among interdisciplinary primary care teams: a mixed-methods feasibility and acceptability trial. Prim Health Care Res Dev. 2019; 20: 1–8. https://doi.org/10.1017/S1463423619000173 PMID: 32799970

84. Ryan RM, Deci EL. Brick by Brick: The Origins, Development, and Future of Self-Determination Theory. Advances in Motivation Science. 2019. pp. 111–156. https://doi.org/10.1016/bs.adms.2019.01.001

85. Lederer AM, Middlestadt SE. Beliefs About Meditating Among University Students, Faculty, and Staff: A Theory-Based Salient Belief Elicitation. Journal of American College Health. 2014; 62: 360–369. https://doi.org/10.1080/07448481.2014.907296 PMID: 24742011

86. van der Riet P, Levett-Jones T, Aquino-Russell C. The effectiveness of mindfulness meditation for nurses and nursing students: An integrated literature review. Nurse Educ Today. 2018; 65: 201–211. https://doi.org/10.1016/j.nedt.2018.03.018 PMID: 29602138

87. Bakker AB, Demerouti E. Job demands–resources theory: Taking stock and looking forward. J Occup Health Psychol. 2017; 22: 273–285. https://doi.org/10.1037/ocp0000056 PMID: 27732008

88. Guidetti G, Viotti S, Badagliacca R, Colombo L, Converso D. Can mindfulness mitigate the energy-depleting process and increase job resources to prevent burnout? A study on the mindfulness trait in the school context. Garbarino S, editor. PLoS One 2019; 14: e0214935. https://doi.org/10.1371/journal.pone.0214935 PMID: 30947256
